# Nutritional and tissue-specific regulation of cytochrome P450 CYP711A *MAX1* homologues and strigolactone biosynthesis in wheat

**DOI:** 10.1093/jxb/erad008

**Published:** 2023-01-10

**Authors:** Petros P Sigalas, Peter Buchner, Stephen G Thomas, Frank Jamois, Mustapha Arkoun, Jean-Claude Yvin, Malcolm J Bennett, Malcolm J Hawkesford

**Affiliations:** Rothamsted Research, West Common, Harpenden AL5 2JQ, UK; Rothamsted Research, West Common, Harpenden AL5 2JQ, UK; Rothamsted Research, West Common, Harpenden AL5 2JQ, UK; Laboratoire de Physico-Chimie et Bioanalytique, Centre Mondial de l’Innovation Roullier, Timac Agro International, 18 Avenue Franklin Roosevelt, Saint-Malo, 35400, France; Laboratoire de Nutrition Végétale, Centre Mondial de l’Innovation Roullier, Timac Agro International, 18 Avenue Franklin Roosevelt, Saint-Malo, 35400, France; Laboratoire de Nutrition Végétale, Centre Mondial de l’Innovation Roullier, Timac Agro International, 18 Avenue Franklin Roosevelt, Saint-Malo, 35400, France; Plant and Crop Sciences, School of Biosciences, University of Nottingham, Sutton Bonington Campus, Loughborough LE12 5RD, UK; Rothamsted Research, West Common, Harpenden AL5 2JQ, UK; CIMMYT, Mexico

**Keywords:** Cytochrome P450 CYP711A, gene expression, MAX1, nitrogen, strigolactones, tillering, wheat (*Triticum aestivum*)

## Abstract

Strigolactones (SLs) are a class of phytohormones regulating branching/tillering, and their biosynthesis has been associated with nutritional signals and plant adaptation to nutrient-limiting conditions. The enzymes in the SL biosynthetic pathway downstream of carlactone are of interest as they are responsible for structural diversity in SLs, particularly cytochrome P450 CYP711A subfamily members, such as *MORE AXILLARY GROWTH1* (*MAX1*) in Arabidopsis. We identified 13 *MAX1* homologues in wheat, clustering in four clades and five homoeologous subgroups. The utilization of RNA-sequencing data revealed a distinct expression pattern of *MAX1* homologues in above- and below-ground tissues, providing insights into the distinct roles of *MAX1* homologues in wheat. In addition, a transcriptional analysis showed that SL biosynthetic genes were systematically regulated by nitrogen supply. Nitrogen limitation led to larger transcriptional changes in the basal nodes than phosphorus limitation, which was consistent with the observed tillering suppression, as wheat showed higher sensitivity to nitrogen. The opposite was observed in roots, with phosphorus limitation leading to stronger induction of most SL biosynthetic genes compared with nitrogen limitation. The observed tissue-specific regulation of SL biosynthetic genes in response to nutritional signals is likely to reflect the dual role of SLs as rhizosphere signals and branching inhibitors.

## Teaser

Expression analysis of strigolactone biosynthetic genes revealed tissue-specific responsiveness to nutritional signals and distinct spatial expression patterns of *MAX1*genes in *Triticum aestivum*.

## Introduction

Nitrogen (N) is one of the primary macronutrients required for plant growth and development. Plant adaptation to N-limiting conditions includes suppression of shoot growth, including suppression of lateral bud outgrowth resulting in a decrease in axillary branching (Luo *et al.*, 2017, 2020). Strigolactones (SLs) are a group of phytohormones that play an essential role in suppressing shoot branching/tillering and also act as a rhizosphere signal (Chesterfield *et al.*, 2020; Mashiguchi *et al.*, 2021). SLs were identified initially as compounds that stimulate the germination of the seeds of the parasitic weed, *Striga* sp, after which they were named (Cook *et al.*, 1966). Later, SLs were linked to the communication between plants and arbuscular mycorrhizal fungi (AMF) (Akiyama *et al.*, 2005). Subsequently, SLs were identified as phytohormones that inhibit shoot branching (Gomez-Roldan *et al.*, 2008; Umehara *et al.*, 2008). Several high branching/tillering mutants, such as *more axillary growth* (*max*) in Arabidopsis (*Arabidopsis thaliana*) and *dwarf* (*d*) in rice (*Oryza sativa*), led to the discovery that SLs play a central role in the modulation of the above-ground plant architecture. Several studies have reported elevated SL levels in root exudates in many species under nutrient-limiting conditions, suggesting that SL production and exudation are linked with nutrient availability such as phosphorus (P) and N (Yoneyama *et al.*, 2007a, b, 2012, 2015; Umehara *et al.*, 2008; Kohlen *et al.*, 2011). Yoneyama *et al.* (2012) reported that N limitation strongly increased the levels of SLs in the roots of many species, including wheat (*Triticum aestivum*). Apart from their role as rhizosphere signals under nutrient limitation facilitating root colonization by AMF, SLs have been suggested to play a role in plant response to nutrient-limiting conditions, acting as a signal coordinating plant growth. It has been demonstrated that SLs are involved in root architectural changes triggered by N- and P-limiting conditions (Sun *et al.*, 2014). Other studies have shown that the increased levels of SLs act as long-distance signals suppressing tillering under P deficiency (Umehara *et al.*, 2010; Kohlen *et al.*, 2011).

**Fig. 1.**
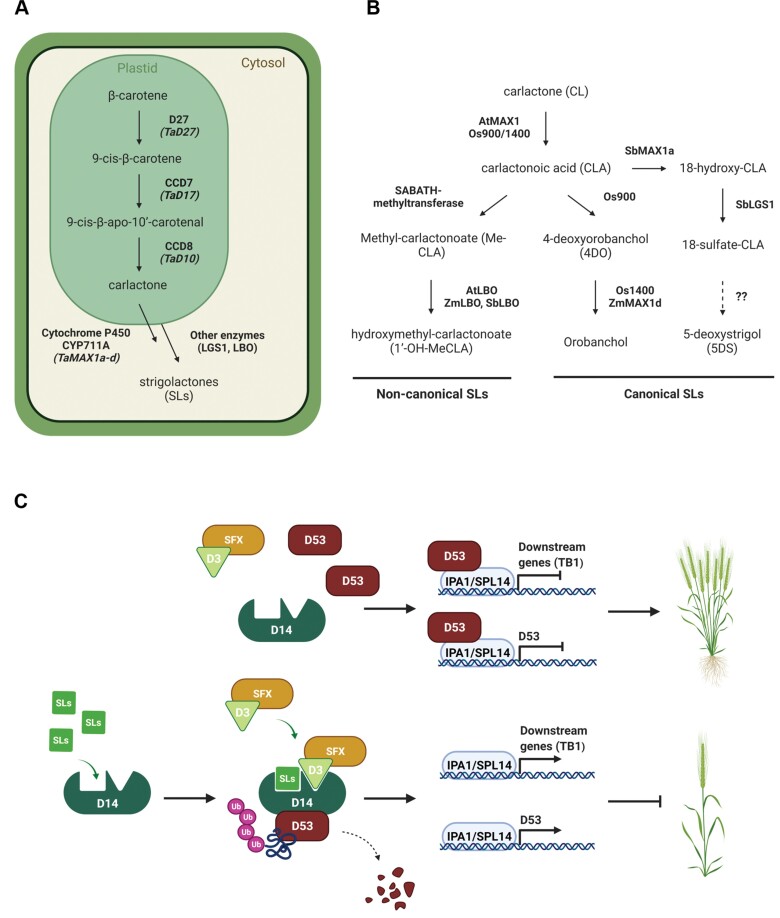
SL biosynthetic and signalling pathways. (A) The compartmentalization of the SL biosynthetic pathway. (B) Steps downstream of CL for the biosynthesis of canonical and non-canonical SLs. (C) SL perception and signalling pathway. Illustration created with BioRender.

SLs are terpenoid lactones derived from the carotenoid biosynthetic pathway (Matusova *et al.*, 2005). Based on their chemical structure, SLs are classified as canonical or non-canonical (Yoneyama and Brewer, 2021). Canonical SLs consist of a four-methyl butanolide ring (D ring) connected with a three-ring structure (ABC) ring, whereas non-canonical SLs lack the ABC ring, which is replaced by other groups. Strigol- or orobanchol-type SLs are canonical SLs found in root exudates, suggesting that they act as rhizosphere signals. The first step of SL biosynthesis is catalysed by β-carotene isomerase, DWARF27 (D27), which catalyses the conversion of all-*trans*-β-carotene into 9-*cis*-β-carotene (Fig. 1A) (Alder *et al.*, 2012). Subsequently, CAROTENOID CLEAVAGE DIOXYGENASE 7 (CCD7) encoded by *AtMAX3* and *OsD17* in Arabidopsis and rice, respectively, catalyses the cleavage of 9-*cis*-β-carotene to 9-*cis*-β-apo-10ʹ-carotenal. The latter is then converted into carlactone (CL) by another member of the carotenoid cleavage dioxygenase family, CCD8 encoded by *AtMAX4* in Arabidopsis and *OsD10* in rice. CL is considered the precursor for the biosynthesis of bioactive SLs. The steps downstream of CL are catalysed by members of the cytochrome P450 CYP711A subfamily. More specifically, in Arabidopsis, CYP711A1 encoded by *AtMAX1* converts CL to carlactonoic acid (CLA) (Abe *et al.*, 2014). Recent studies have also revealed enzymes downstream of CLA (Fig. 1B). Wakabayashi *et al.* (2021) found that a SABATH methyltransferase catalyses the conversion of CLA to Me-CLA in Arabidopsis. Me-CLA is a non-canonical SL which interacts with D14, the receptor protein of the SL signalling pathway, indicating that it is biologically active in suppressing shoot branching (Abe *et al.*, 2014). Me-CLA is used as a substrate by LATERAL BRANCHING OXIDOREDUCTASE (LBO), which converts Me-CLA to hydroxymethyl-carlactonoate (1ʹ-OH-MeCLA) in Arabidopsis, while the function of LBO was found to be conserved in both maize (*Zea mays*) and sorghum (*Sorghum bicolor*) (Brewer *et al.*, 2016; Yoneyama *et al.*, 2020a). However, there are multiple *MAX1* genes present in monocotyledon genomes, whose functions remain an important topic. In fact, five different *MAX1* homologues have been found in the rice genome, while maize and sorghum have three and four homologues, respectively (Yoneyama *et al.*, 2018). Studies in those species have shown that different CYP711A/MAX1 proteins catalyse different steps downstream of CL and might be responsible for the large structural diversity of bioactive SLs found in plants (Fig. 1B) (Yoneyama *et al.*, 2018; Wu and Li, 2021). In rice, CLA is an intermediate for the conversion of CL to 4-deoxyorobanchol (4DO) by Os900 (CYP711A2), while Os1400 (CYP711A3) converts 4DO to orobanchol (Zhang *et al.*, 2014; Yoneyama *et al.*, 2018). Less is known about the function of other CYP711A/MAX1 proteins in rice, such as Os1900 and Os5100. More recently, a new biosynthetic pathway of canonical SL biosynthesis was identified in sorghum. More specifically, SbMAX1a was found to convert CL to 18-hydroxy-CLA. The latter is used as a substrate by a sulfotransferase encoded by *LOW GERMINATION STIMULANT 1*, which is probably involved in the biosynthesis of 5-deoxystrigol (5DS) and 4DO (Wu and Li, 2021). Recent studies have provided insights into the phylogeny of *MAX1* genes in monocotyledons, but most of the studies either did not include wheat sequences or did not include all *MAX1* genes present in the wheat genome (Yoneyama *et al.*, 2018; Marzec *et al.*, 2020; Wu and Li, 2021; Vinde *et al.*, 2022).

*D14* encodes an α/β-hydrolase protein, which acts as the receptor protein of SL signals (Yao *et al.*, 2016; Shabek *et al.*, 2018). In the presence of SLs, D14 interacts with F-box protein D3 (MAX2 in Arabidopsis). D3 is part of the Skp1–Cullin–F-box (SCF) E3 ubiquitin ligase complex (SCF^D3^) responsible for the ubiquitination and proteasomal degradation of targeted proteins. In rice, the D53 nuclear repressor protein has been shown to be the target of the D14–SCF^D3^ complex (Zhou *et al.*, 2013). Therefore, in the presence of SLs, D53 is ubiquitinated and degraded, and the expression of downstream genes repressed by D53 is released (Fig. 1C). Similarly, in Arabidopsis, SL signalling relies on the degradation of SUPPRESSOR OF MAX2 1-LIKE6, 7, and 8 (SMXL6, 7, 8), which are the functional orthologous proteins of D53 (Soundappan *et al.*, 2015; Wang *et al.*, 2015). D53 repressor has been found to control its own transcription, forming a negative feedback loop regulation. Thereby, in the presence of SLs, *D53* transcription is induced, whereas, in the absence of SLs, *D53* is suppressed (Fig. 1C) (Song *et al.*, 2017; Wang *et al.*, 2020). Among the genes targeted by the SL signalling pathway is the gene encoding the transcription factor TEOSINTE BRANCHED 1 (TB1), which is known to act as a negative regulator of tillering/branching, acting as an integrator of environmental and developmental signals (Takeda *et al.*, 2003; Aguilar-Martínez *et al.*, 2007; Liu *et al.*, 2017; Song *et al.*, 2017).

Although it has been demonstrated that SL production is linked with nutrient availability, few studies, mainly in rice, have focused on the transcriptional regulation of SL biosynthetic and signalling genes in response to N availability (Sun et al., 2014, 2021; Xu *et al.*, 2015); there are no extensive studies on SL-related gene regulation in wheat. In addition, even in well-studied monocotyledonous species, transcriptional studies have mainly focused on the genes involved in the core SL biosynthetic pathway, such as *D27*, *D17*, and *D10*, whereas *MAX1* genes are rarely included (Xiu-mei *et al.*, 2015). In addition, less is known about the signals controlling SL biosynthesis under N limitation. SL production and exudation are mainly associated with P limitation and, to a lesser extent, with N limitation (Yoneyama *et al.*, 2012). Therefore, SL up-regulation under N-limiting conditions has been attributed to the lower P uptake by N-limited plants rather than directly to plant N status. In split-root experiments, it has been demonstrated that neither N nor P itself controls SL biosynthesis in sorghum and rice under N- or P-limiting conditions, respectively. As a result, it has been speculated that shoot-derived signals of N and P status might be responsible for SL production regulation (Yoneyama *et al.*, 2015, 2020b), but this regulation remains elusive, especially in wheat.

The aim of this work was to identify wheat SL biosynthetic and signalling genes and study their transcriptional regulation by N supply. For that reason, gene expression of most of the identified SL-related genes was monitored in response to N limitation in hydroponically grown wheat in both the root and the basal nodes. In addition, by utilizing data from different RNA-sequencing (RNA-seq) experiments, the spatial expression patterns of *MAX1* homologues were examined, providing some indications of the tissue-specific expression and the functionality of the different *MAX1* homologues present in the wheat genome. A comparative analysis of the P and N limitation effects on the regulation of SL-related gene expression was performed, showing that P limitation had a stronger effect on SL biosynthesis in roots, whilst N limitation led to a more substantial induction of SL biosynthesis in the basal nodes.

## Materials and methods

### Identifying wheat orthologous genes

Wheat orthologous genes of interest were identified from the protein sequences from rice, Arabidopsis, and barley (*Hordeum vulgare*) (Zhang *et al.*, 2014; Liu *et al.*, 2017; Kobae *et al.*, 2018; J. Wang *et al.*, 2018). The amino acid sequences obtained were used as queries against the IWGSC RefSeq Annotation v1.1. using the BLASTP tool on EnsemblPlants (Kersey *et al.*, 2018). The protein sequences with the highest homology from the A, B, and D genomes were identified. For further confirmation of homology, the wheat sequences obtained were used for reciprocal BLASTP back to rice protein databases. The accession number of the identified SL-related genes can be found in Supplementary Table S1.

### Sequence alignment and phylogenetic analysis

Multiple sequence alignment was performed using MUSCLE with the default parameters (Edgar, 2004), as implemented in Geneious Prime® 2022.2.2 (https://www.geneious.com/). The multiple protein sequence alignment was used for the construction of the phylogenetic tree based on the Neighbor–Joining method (Nei and Saitou, 1987) using Geneious Tree Builder. Bootstrap values for the trees were calculated as a percentage of 1000 trials.

### PCR and DNA sequencing

For sequencing of the ambiguous region upstream of *TaMAX1a2-3B*, PCR was performed with a Touchgene Gradient PCR machine (Techne, Staffordshire, UK), using Q5 high-fidelity DNA polymerase (New England BioLabs, Ipswich, MA, USA) following the manufacturer’s protocol and homoeologue-specific primers (Forward primer, GATTGTGGACTAATGACCGTGATTG; Reverse primer, TAGAAGTGCTTTTCGATGAAATCC; annealing temperature, 64 °C). After the amplification of the targeted region, PCR products were purified with the Wizard SV Gel and PCR Clean-Up System (Promega, Madison, WI, USA), following the manufacturer’s protocol. The PCR products were sequenced using the TubeSeq service (Eurofins Genomics, Luxemburg, Luxemburg).

### Hydroponic culture of wheat plants

For the hydroponic experiments, *T. aestivum* cv. Cadenza plants were cultured in a custom hydroponic system in a controlled-environment chamber. Seeds were surface sterilized with 1:40 bleach solution:dH_2_O (v/v) for 15 min, followed by five washes with sterilized dH_2_O. Seeds were soaked in sterilized dH_2_O overnight at 4 °C in the dark. Subsequently, seeds were placed in black boxes with wet filter paper to germinate (Day 0). Individual plants were grown held by foam buds on top of 1 litre black pots containing the nutrient solution. The nutrient solution was aerated throughout the experiment by an aeration pump tubing system. The growing conditions were 20/10 °C day and night temperature, respectively, and 14 h day length. Lighting was provided by fluorescent bulbs at an intensity of 550 µmol m^–2^ s^–1^. The humidity was stable at 65% during the day and 75% during the night. Seedlings were transferred to the hydroponic culture system 4 days after sowing (DAS). Plants were supplied with half-strength nutrient solution for 3 d (until 7 DAS) and then supplied with full-strength nutrient solution until sampling. The composition of the basic nutrient solution, modified Letcombe (Drew and Saker, 1984), was 1.5 mM Ca(NO_3_)_2_, 5 mM KNO_3_, 2 mM NaNO_3_, 1.5 mM MgSO_4_, 1 mM phosphate buffer solution (KH_2_PO_4_–K_2_HPO_4_, pH 6.0), 50 μM FeNaEDTA, 0.5 μM CuCl_2_, 20 μM H_3_BO_3_, 3.6 μM MnCl_2_, 0.1 μM Na_2_MoO_4_·2H_2_O, and 0.77 μM ZnCl_2_. In addition, 2.35 mM MES monohydrate was added as a buffer and the pH was adjusted to 5.8 with KOH. The nutrient solution was renewed every 3–4 d. For low N treatment, plants were supplied with 0.1 mM N in the form of KNO_3_. For ionic balance in the low N nutrient solution, Ca(NO_3_)_2_ was replaced by CaCl_2_, KNO_3_ by KCl, and NaNO_3_ by NaCl. Similarly, in the low P treatment, plants were supplied with 0.01 mM KH_2_PO_4_, while the remaining amount of KH_2_PO_4_ was replaced by KCl. In the second experiment, P was supplied only as KH_2_PO_4_ in all the treatments instead of the phosphate buffer solution.

### Experimental design

To determine the effect of N limitation on the transcription of the genes of interest at different time points, a complete randomized block design with three blocks and three biological replicates per treatment combination was used. All plants received full-strength nutrient solution until 10 DAS, when they were divided into two N treatments, high N (10 mM N) and low N (0.1 mM N). The first sampling was conducted at 10 DAS and before dividing plants into the two treatments. High N- and low N-treated plants were sampled 4, 8, and 12 d after the N limitation (14, 18, and 22 DAS). The selection of the time points was based on previous experimentation showing that those dates corresponded to the dates when the first, second, third, and fourth tiller bud of the main stem starts to actively grow under the given growing conditions. Each biological replicate consisted of pooled tissue material from four plants to ensure enough material for subsequent analysis.

For the N and P limitation experiment, a complete randomized block design with four biological replicates per treatment was used. All plants received full-strength nutrient solution until 10 DAS, when they were divided into the three treatments, control (10 mM N, 1 mM P), low N (0.1 mM N, 1 mM P), and low P (10 mM N, 0.01 mM P). The sampling took place at 18 DAS, 8 d after the introduction of plants to the respective nutrient limitation. Each biological replicate consisted of the material from six plants pooled together to ensure enough material for subsequent analysis.

### Tissue harvesting/sampling

The whole root system, the basal node, and the remaining shoot and leaves were harvested at specific time points based on each experimental design. In this study, the basal node was defined as the 0.5 cm long section from the base of the main shoot, which includes the shoot apical meristem, lateral buds, and leaf meristems. After removing all the roots from the shoot, the outgrown primary tillers were also removed, and 0.5 cm of the main shoot base was dissected. Samples were quickly frozen in liquid nitrogen and stored at –80 °C before processing.

### RNA extraction

Tissue samples were ground to a fine powder in liquid nitrogen. Basal node samples were hand-ground with a pre-cooled mortar and pestle due to the small amount of the sampled tissue. Grinding of the root samples was carried out in Freezer Mill (SPEX SamplePrep, Metuchen, NJ, USA) following the manufacturer’s instructions.

For basal node samples, total RNA was extracted from ~100 mg of frozen tissue using TRIzol™ (Invitrogen, Waltham, MA, USA) reagent, followed by DNase treatment with RQ1 RNase-Free DNase (Promega, Madison, WI, USA) to ensure no DNA carryover following the manufacturer’s protocols. Subsequently, RNA was further purified by phenol/chloroform/isoamyl alcohol purification. RNA was precipitated by adding 3 vols of absolute EtOH and 0.1 vols of 3 M NaOAc (pH 5.2) and incubation at –80 °C for at least 1 h. A modified hot phenol extraction protocol was used for extracting RNA from ~500 mg of root tissue (Verwoerd *et al.*, 1989). The DNase treatment, the phenol/chloroform/isoamyl alcohol purification steps, and the RNA precipitation were conducted as described above. All the solutions used for the RNA extractions had been previously treated with diethyl pyrocarbonate to destroy any RNase activity.

Total RNA concentration was measured using a Nanodrop 2000 spectrophotometer (Thermo Scientific, Waltham, MA, USA), while *A*_280_/*A*_260_ was used as a quality check of the extracted RNA. The quality of the extracted RNA was also evaluated by running 500 ng of RNA in a 1% agarose TAE gel.

### Quantitative reverse transcription–PCR (RT–qPCR)

First-strand cDNA was synthesized from 2 μg of total RNA using SuperScript III Reverse Transcriptase (Invitrogen, Waltham, MA, USA) and oligo(dT) primers following the manufacturer’s instructions. Synthesized cDNA was diluted 1:1 with RNase-free H_2_O and stored at –20 °C.

The real-time qPCRs were performed in an ABI-7500 real-time PCR system (Applied Biosystems, Waltham, MA, USA) using SYBR® Green JumpStart™ Taq ReadyMix™ (Sigma-Aldrich, Dorset, UK). The 20 μl reactions contained 1 μl of cDNA and 250 nM of each primer. A pipetting scheme for the preparation of each reaction was followed to minimize pipetting mistakes and ensure repeatable results. Each reaction was initially prepared in a 0.5 ml microcentrifuge tube by adding 21.4 μl of mastermix (11.25 μl of SYBR Green JumpStart Taq ReadyMix, 0.6 μl of 10 μM Forward Primer, 0.6 μl of 10 μM Reverse Primer, 0.02 μl of ROX, and 8.93 μl of H_2_O_DEPC_) and 1.1 μl of the cDNA sample. After mixing the reactions and a short centrifugation, 20 μl of each reaction were pipetted into a white 96-well PCR Plate (4titude, Surrey, UK), which was sealed with an adhesive qPCR seal (4titude, Surrey, UK). The real-time PCR cycling parameters used were 2 min at 50 °C and 10 min at 95 °C, followed by 40 cycles of 95 °C for 15 s and 60 °C for 60 s. At the end of the reaction, the melting curve was recorded.

For gene expression analysis by RT–qPCR, primers were designed that amplify all the three homoeologues of the genes of interest. The primer design tool, Primer 3 v2.3.7, as implemented in Geneious 10.2.3 (https://www.geneious.com), was used for designing primers, preferably close to the 3ʹ end of the coding sequence. In some cases, the primers were designed manually. The online tool OligoCalc (Kibbe, 2007) was used for the calculation of the primer characteristics.

Each set of designed primers (forward and reverse) was tested for specificity and efficiency of the amplification by using a dilution series of template cDNA from different plant tissues (shoots and roots). The efficiency of each set was calculated based on the slope of the standard curve, and the melting curve was used for evaluating the primer specificity. Primers that showed high primer efficiency and a melting curve with a single peak in a range of template cDNA were selected (Supplementary Table S2).

### RT–qPCR data analysis

The real-time qPCR data were analysed with ABI 7500 v2.0.5 software. The fluorescence threshold for calculation of the Ct values was calculated automatically by the software or manually adjusted in the linear phase of the amplification curve. Primer efficiency was estimated for all individual samples in each run using the linear phase of the amplification curves as calculated by the LinRegPCR software (Ramakers *et al.*, 2003).

The gene expression levels were calculated as normalized relative quantity (NRQ) given by the formula $\text{NRQ=}\frac{{\left({\text{E}}_{\text{GOI}}\right)}^{\text{-Ct;GOI}}}{\sqrt[{\text{2}}]{{\left({\text{E}}_{\text{ref1}}\right)}^{\text{-Ct;ref1}}\times {\left({\text{E}}_{\text{ref2}}\right)}^{\text{Ct;ref2}}}}$ (Rieu and Powers, 2009). Ct_GOI_ and E_GOI_ stand for the Ct value and the average primer efficiency of the targeted gene of interest, respectively. Similarly, Ct_ref_ and E_ref_ correspond to the Ct value and the primer efficiency of the two reference genes. Wheat actin3 (*TaACT3*) and succinate dehydrogenase (*TaSDH*) genes were used as reference genes (Supplementary Table S2). Two technical replicates were used for each of the reference genes.

### High-throughput RNA-sequencing

Extracted total RNA from basal node samples was further purified with the Plant RNeasy kit (Qiagen, Hilden, Germany) following the provided RNA clean-up protocol. The concentration of the purified RNA was determined by the Qubit Broad Range assay (Invitrogen, Waltham, MA, USA). A 2–3 μg aliquot of RNA samples was submitted for next-generation sequencing (NGS).

Prior to the library preparation, RNA integrity was assessed with the RNA kit on an Aligent 5300 Fragment Analyzer. Quality control, poly(A) selection for rRNA removal, library preparation, multiplexing, and sequencing were performed by Genewiz UK according to their standard workflow. Briefly, RNA library preparation was prepared using the NEBNext Ultra II Library Prep Kit for Illumina, following the manufacturer’s protocol. Firstly, oligo(dT) beads were used for mRNA enrichment, and the mRNAs were fragmented for 15 min at 94 °C. First- and second-strand cDNAs were subsequently synthesized. Indexed adapters were ligated to cDNA fragments after adenylation of the 3ʹ end of the cDNA fragments. Limited cycle PCR was used for library amplification. Sequencing libraries were validated using the NGS Kit on the Agilent 5300 Fragment Analyzer and quantified by using a Qubit 4.0 Fluorometer or equivalent. The sequencing libraries were multiplexed and loaded on the flow cell. NGS was performed in Illumina Novaseq 6000 with 2 × 150 bp pair-end configuration v1.5. Image analysis and base calling were conducted by the NovaSeq Control Software v1.7 on the NovaSeq instrument. Raw paired-end data were delivered in fastq format after de-multiplexing and standard adapter trimming by the sequencing contractor. One mismatch was allowed for index sequence identification.

### RNA-sequencing data analysis workflow

All the tools used for the RNA-seq data analysis were executed in the Rothamsted Research Galaxy platform (https://galaxy.rothamsted.ac.uk/). The RNA-seq raw data were received in fastq format. The FastQC v0.11.7 tool was used to assess the quality of the raw data (http://www.bioinformatics.babraham.ac.uk/projects/fastqc/). If required, low-quality reads and adapter sequences were removed using Cutadapt v3.7 (Martin, 2011). Subsequently, trimmed reads were pseudo-aligned to *T. aestivum* IWGSC RefSeq v1.0 annotation v1.1 using kallisto v0.46.0.4 (Bray *et al.*, 2016) for calculating transcript abundance reported as transcripts per million mapped reads (TPM). The package tximport v1.24.0 was used to create gene-level count matrices from the transcript abundances (Soneson *et al.*, 2015). The DESeq2 v1.32.0 package (Love *et al.*, 2014) was used to perform the differential gene expression analysis (alpha=0.01) by fitting the appropriate model based on the experimental design. A pre-filtering of low count genes was performed before performing differential gene expression analysis. Only genes that had at least four samples with more than five reads were included in the analysis. Significantly differentially expressed genes were retrieved by applying post-hoc filtering of *P*adj<0.01 and |log_2_fold change (FC)|>0.58 (|FC|>1.5). The DESeq2 package was run in R Statistical Software v4.1.1 on a local Windows machine. For the visualization of the RNA-seq data analysis, the pheatmap v1.0.12 package was used in R Statistical Software v4.1.1.

TPM values were also used for comparing the gene expression levels of different genes since this value is normalized for both gene length and sequencing depth, allowing the comparison between different genes.

### RNA-seq validation

RNA-seq results were also confirmed by comparing the gene transcript abundance (TPM) with the relative gene expression value (NRQ) obtained from RT–qPCR. Three different genes were used for the RNA-seq validation, *TaD27*, *TaD17*, and *TaCKX3* (TraesCS1A02G159600, TraesCS1B02G176000, and TraesCS1D02G157000). Pearson correlation between transcript abundance values of selected genes obtained from the RNA-seq and RT–qPCR was performed using package ggpubr v0.4.0 in R statistical software v4.1.1. For all three genes, there was a good correlation (*R*>0.9) between the expression values obtained from the RNA-seq and the RT–qPCR, while the effect of the treatment was also found to be consistent in both methods, suggesting that the RNA-seq data were reliable (Supplementary Fig. S1).

### Expression analysis of *MAX1* homologues from RNA-seq data

For examining the expression pattern of the identified *MAX1* genes, apart from the RNA-seq performed as part of this study in basal nodes of wheat plants (cv Cadenza), additionally publicly available RNA-seq data were utilized. The first study (ArrayExpress accession: E-MTAB-11927) included two different tissues, root and basal nodes, of N-stressed hydroponically grown wheat plants (cv Graham) with three replicates. The second study (BioProject: PRJNA749278, SRA: SRP329634) was performed on the root and shoot of four different wheat cultivars (cv big sky, cv OK1059060, cv OCW00S063S-1B, and cv NF97117) grown under sufficient or low N supply. This experiment was conducted in pots under glasshouse conditions with three replicates per treatment. Finally, RNA-seq from previously published work (BioProject: PRJDB2496, SRA: DRP000768) on P-starved wheat (cv Chinese Spring) roots and shoots was utilized (Oono *et al.*, 2013). All the raw data were downloaded from the NCBI SRA database (the list of samples can be found in Supplementary Table S3). Kallisto v0.46.0.4 (Bray *et al.*, 2016) was used for quantifying the transcript abundance using IWGSC RefSeq v1.0 annotation v1.1.

### Chemical analysis

The whole root and shoot samples were ground into a fine powder using a Freezer Mill (SPEX SamplePrep) following the manufacturer’s instructions. Samples were then lyophilized in a Modulyo freeze dryer (Edwards, West Sussex, UK) for at least 48 h or until thoroughly dried, and then submitted for chemical analysis. The total N content of dried root or shoot samples was measured by the LECO CN628 combustion analyser following the manufacturer’s protocol. For elemental analysis, root and shoot samples were first digested using 85:15 (v/v) nitric acid:perchloric acid in an open tube digestion block. After volatilization of the acids, the residues were dissolved in 5% (v/v) nitric acid. Sample analysis was carried out with an Agilent 5900 SVDV inductively coupled plasma-optical emission spectrometer (ICP-OES).

### Statistical analysis

Mean values and SEs were calculated from at least three biological replicates, depending on the experimental design. The exact number of biological replicates is mentioned in each figure legend. The statistically significant effect of the treatments/factors was assessed with an ANOVA. Statistical analysis was performed in GenStat statistical software package (21st edition) or by using the package rstatix v0.7.0 in R Statistical Software v4.1.1.

ANOVA was applied to all single variate data, including phenotypic data (tiller counting), gene expression data, and chemical analysis data. Repeated measured ANOVA was conducted for tillering data recorded at different time points in the same individuals. Prior to ANOVA, assumptions for normality and homogeneity of variances were assessed using Shapiro–Wilk’s normality test and Levene’s test, respectively. Following the ANOVA, Fisher’s least significant difference (LSD) at 5% (*P*=0.05), calculated based on the SE of the difference between means on the residual degrees of freedom from the ANOVA, was used to compare relevant group means. Relevant significant comparisons are reported in the figures. In cases where the assumption of homogeneity of variance was not met, Welch’s ANOVA followed by Games–Howell post-hoc test were used instead.

For the gene expression data by RT–qPCR, the Ctʹ values were used for the statistical analysis,${\text{Ct}}^{\text{'}}{\text{=log}}_{\text{2}}\left(\frac{\text{1}}{\text{NRQ}}\right)$,rather than the NRQ. The NRQ data are not linear, and the variability across the treatments is typically too high, so the transformation of the data is required (Rieu and Powers, 2009).

Figures and graphs were created in GraphPad Prism v9.3.1 for Windows or by using ggplot2 v3.3.5 in R Statistical Software v4.1.1. For the generations of heatmaps based on the RNA-seq data, the package pheatmap v1.0.12 was used.

## Results

### Identification of SL-related genes in hexaploid wheat

The initial step for studying the response of SL-related genes to nutritional signals in wheat was to identify the wheat orthologues involved in SL biosynthesis, perception, and signalling (Fig. 1). For the identification of D27, the first enzyme of the SL biosynthetic pathway, blastp revealed three amino acid sequences with high similarity, sharing ~70% similarity to OsD27 (Os11t0587000) (Supplementatry Table S4). The wheat sequences obtained are encoded by homoeologous genes located on chromosomes 7A, 7B, and 7D, and they showed high similarity to the previously identified HvD27 in barley (H. Wang *et al.*, 2018). Therefore, TraesCS7A02G418900, TraesCS7B02G319100, and TraesCS7D02G411500 were assigned as *TaD27-7A*, *TaD27-7B*, and *TaD27-7D*, respectively.

The second and third steps of SL biosynthesis are catalysed by CCD7 and CCD8 enzymes, members of the CCD family, which in rice are encoded by *OsD17* (Os04g0550600) and *OsD10* (Os01g0746400), respectively. Three homoeologous coding sequences were identified in chromosomes 2A (TraesCS2A02G414600), 2B (TraesCS2B02G433800), and 2D (TraesCS2D02G411900), which encode proteins sharing ~77% similarity to *OsD17* (Supplementary Table S4). In contrast, six protein sequences were most closely related to OsD10 (Supplementary Table S4). The phylogenetic analysis revealed that all the identified wheat sequences were grouped closely to *OsD10* and *HvD10* and were divided into two distinct homoeologous subgroups (Supplementary Fig. S2). The protein sequences encoded by TraesCS3A02G274300, TraesCS3B02G308000, and TraesCS3D02G273500 were grouped together forming subgroup a; therefore, they were designated as *TaD10a-3A*, *TaD10a-3B*, and *TaD10a-3D*, respectively. The second *D10* wheat subgroup (b) consists of two genes located on chromosome 3A (TraesCS3A02G074200 andTraesCS3A02G074100) and one on chromosome 3B (TraesCS3B02G088400); consequently, they were considered to be *TaD10b* genes. Based on publicly available wheat gene expression data available on Wheat eFP Browser (Ramírez-González *et al.*, 2018), *TaD10b* homoeologues were not expressed in most tissues, whereas *TaD10a* homoeologues were expressed in multiple tissues (Supplementary Fig. S3). Thus, only sequences of *TaD10a* homoeologues were considered for the gene expression analysis in this study.

For the identification of the wheat orthologues of the two main components of the SL perception pathway, the protein sequences of OsD3 (Os06t0154200) and OsD14 (Os03t0203200) were used as templates. The wheat proteins encoded by TraesCS7D02G106000, TraesCS7B02G008400, and TraesCS7A02G110500 showed the highest identity to OsD3 (~70%) (Supplementary Table S4), hence they were assigned as *TaD3-7D*, *-7B*, and *-7A*, respectively. Similarly, the proteins encoded by TraesCS4A02G046700, TraesCS4B02G258200, and TraesCS4D02G258000 shared >86% protein similarity with OsD14 and were assigned as the putative *TaD14* genes (Supplementary Table S4).

In rice, two genes have been identified encoding D53, the main repressor of the SL signalling pathway: *OsD53* (Os11t0104300) and *OsD53-like* (Os12g0104300). In Arabidopsis, AtSMXL6 (AT1G07200), AtSMXL7 (AT2G29970), and AtSMXL8 (AT2G40130) are considered as orthologues of OsD53. In total, six wheat genes were retrieved encoding proteins with ~70% similarity to both rice D53 proteins (Supplementary Table S4). The phylogenetic analysis showed that the identified wheat sequences form two distinct homoeologous subgroups (Supplementary Fig. S4). Therefore, TraesCS4A02G182800, TraesCS4B02G135800, and TraesCS4D02G130600 were assigned as *TaD53a*. Similarly, TraesCS5A02G155000, TraesCS5B02G153200, and TraesCS5D02G159900 were named *TaD53b*. Based on their expression pattern, all six identified putative wheat *TaD53* genes were found to be expressed in various tissues based on publicly available wheat gene expression data.

### Identification and phylogenetic relationship of hexaploid wheat *MAX1* homologues

**Fig. 2.**
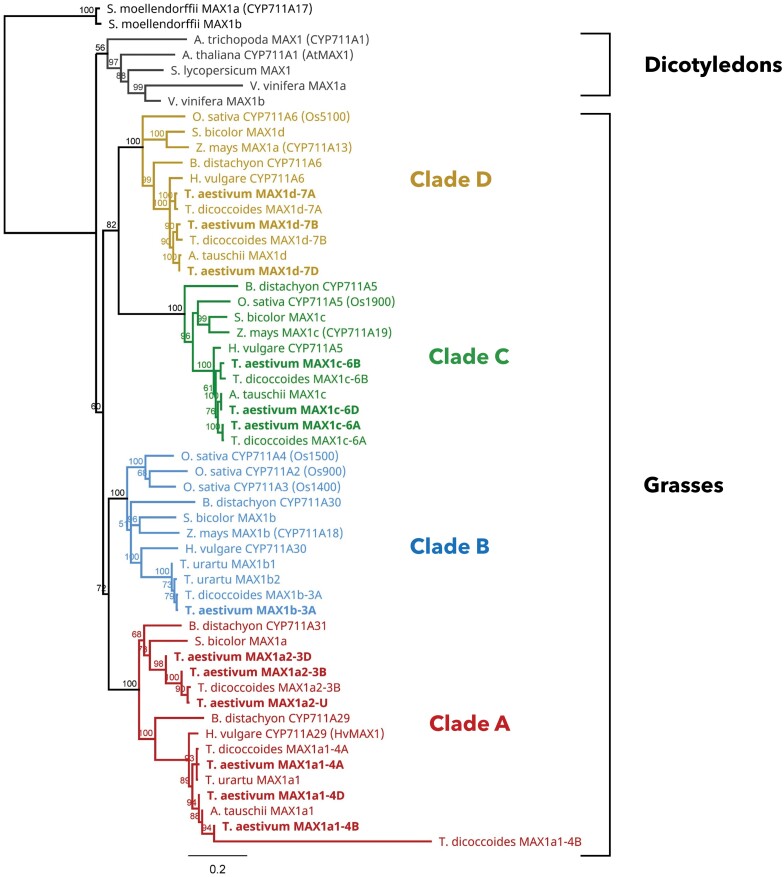
Phylogenetic relationship of CYP711A/MAX1 proteins mainly from grasses such as hexaploid wheat, rice, barley, sorghum, maize, and *B. distachyon*, from dicotyledons such as Arabidopsis, *Solanum lycopersicum*, *Vitis vinifera*, and *Amborella trichopoda*, and from the lycophyte *Selaginella moellendorffii*. The protein sequences of wheat progenitors; *Triticum urartu* (AA), *Triticum dicoccoides* (AABB), and *Aegilops tauschii* (DD) were also included. The tree was constructed using MUSCLE sequence alignment and the Neighbor–Joining method. The different colours (red, blue, green, and yellow) correspond to the four different clades of CYP711A/MAX1 identified in grasses (A–D). The bootstrap values, expressed as a percentage, were obtained from 1000 replicates. The accession number of the sequences used in the analysis can be found in Supplementary Table S5 and the protein alignment in Supplementary Fig. S5.

Monocotyledonous species have multiple *MAX1* homologues present in their genome (Yoneyama *et al.*, 2018; Marzec *et al.*, 2020; Vinde *et al.*, 2022). Based on homology searches using the five rice MAX1 proteins (Os1500, Os5100, Os1900, Os900, and Os1400), 13 distinct amino acid sequences were found in hexaploid wheat (Supplementary Table S1). A phylogenetic analysis was conducted to understand the relationship of the identified proteins, which included 48 sequences from grasses such as rice, maize, barley, sorghum, and *Brachypodium distachyon*. Orthologous sequences of wheat progenitors *Triticum urartu*, *Triticum turgidum* subsp *dicoccoides*, and *Aegilops tauschii* were also included. In addition to grasses, MAX1 sequences from representative dicotyledonous species and one lycophyte were also included (Supplementary Table S5). The phylogenetic analysis revealed that MAX1 from grasses formed four clades (A–D) (Fig. 2; Supplementary Fig. S5); therefore, wheat *MAX1* homologues were named after the clade they fall into (*MAX1a–MAX1d*). Six different wheat sequences were clustered to clade A, forming two distinct subgroups. The first subgroup of clade A consisted of three genes located on chromosome 4 (TraesCS4A02G412100, TraesCS4B02G312300, and TraesCS4D02G309900) along with BdCYP711A29 and HvCYP711A29 (MAX1). The second subgroup of clade A consists of TraesCS3B02G088700-, TraesCS3D02G073900-, and TraesCSU02G146300-encoded proteins along with SbMAX1a and BdCYP711A31. Noticeably, no wheat homologue was identified on chromosome A in clade A2. ­Consistently, neither *T. urartu*, the progenitor of the wheat A genome, nor *T. dicoccoides* was found to have a homologue on chromosome A in clade A2. The genes of clade A which fall into separate subgroups were named *MAX1a1* and *MAX1a2*. In addition, it is worth mentioning that only sequences from wheat and *B. distachyon* were found in both subgroups of clade A, whereas none of the known rice sequences was found in clade A. On the contrary, only TraesCS3A02G466400 was present in clade B, along with three of the rice MAX1 homologues (Os1500, Os900, and Os1400) and sequences from other grasses. No *MAX1b* homologue was found in *T. dicoccoides* chromosome B or *A. tauschii*, supporting the absence of Ta*MAX1b* homoeologues in chromosome B and D. Clade C consists of three wheat orthologues of Os1900 located on chromosome 6 (TraesCS6A02G187200, TraesCS6B02G217300, and TraesCS6D02G174100). Finally, the protein sequences encoded by TraesCS7A02G360300, TraesCS7D02G362800, and TraesCS7B02G267500 fall into clade D along with Os5100.

The majority of the identified wheat MAX1 proteins had lengths >500 amino acids, suggesting that they are functional MAX1s, on the basis that the average length of CYP711A/MAX1 was 515 amino acids. However, *TaMAX1a2-3B* encodes a protein of only 300 amino acids (Supplementary Fig. S5; Supplementary Table S5). Further analysis showed that *MAX1a2-3B* from *T. dicoccoides* encodes a protein of 540 amino acids, suggesting a functional homologue. *TdMAX1a2-3B* has five exons and a coding region of 1623 bp, while *TaMAX1a2* consists of three exons and a coding region of just 939 bp (Supplementary Fig. S6). By exporting the genomic sequence of *TaMAX1a2-3B*, an ambiguous region of >1.5 kb was found upstream of the annotated start codon. Sequencing of that region revealed that this region is homologous to the sequence of *TdMAX1a2-3B* and corresponds to the missing exons, suggesting that *TaMAX1a2-3B* in fact encodes a functional MAX1 homologue. *TaMAX1a2-3D* also encodes a protein of 216 amino acids; however, analysis of the genomic location did not reveal any problem with the genome assembly as in the case of *TaMAX1a2-3B*, suggesting that *TaMAX1a2-3D* may not encode a functional MAX1 homologue.

### Expression pattern of wheat *MAX1* homologues

**Fig. 3.**
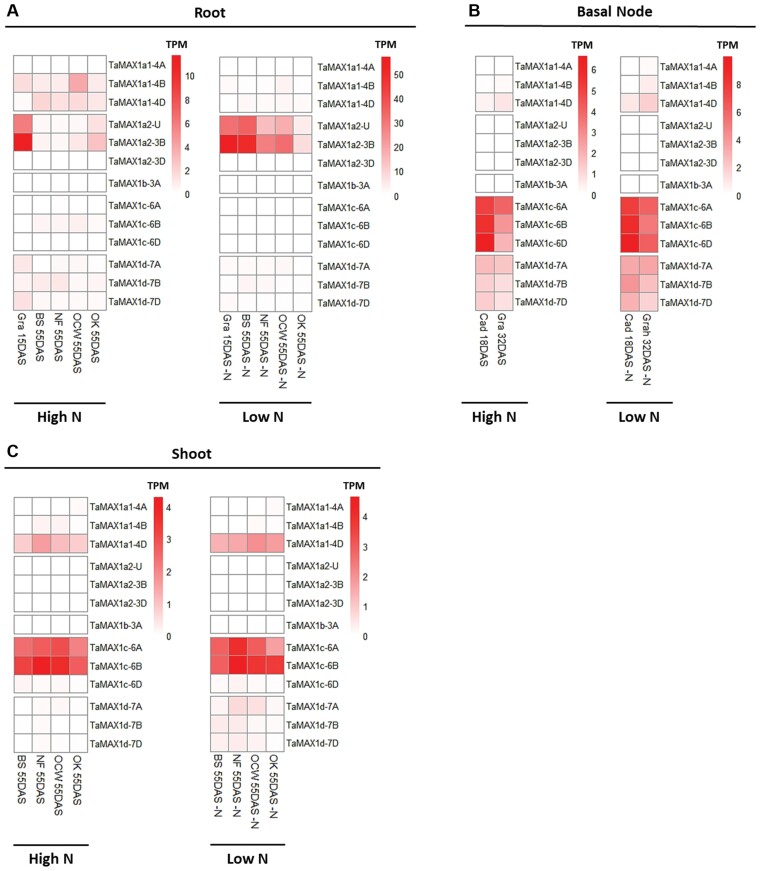
Expression pattern of *TaMAX1* homologues in different tissues. Heatmap comparison of *TaMAX1* gene expression values expressed as TPM in (A) the root, (B) the basal nodes, and (C) the shoot. Data are means of at least three biological replicates. Data were retrieved from different RNA-seq experiments (Supplementary Table S3). Gra, cv Graham; BS, cv big sky; NF, cv NF97117; OCW, cv OCW00S063S-1B; OK, cv OK1059060; Cad, cv Cadenza; DAS, days after sowing.

Data from different RNA-seq experiments were utilized to compare the transcript abundance of the identified wheat *MAX1* homologues in three different tissues. More specifically, the data were obtained from four independent experiments conducted in either root, basal node, or shoot tissues of different wheat cultivars, including samples from nutrient-stressed plants. TPM values of all the 13 identified wheat *MAX1* genes were retrieved and are presented as a heatmap in Fig. 3 (Supplementary Fig. S7; Supplementary Table S6). Genes with TPM values <0.5 were considered as expressed at a low level. Based on the heatmap, there is a distinct pattern of *TaMAX1* expression distinguishing roots, basal nodes, and shoots. *TaMAX1a2-U* and *TaMAX1a2-3B* are the predominant *TaMAX1* genes expressed in the root, showing the highest expression values among all of the *TaMAX1* homologues (Fig. 3A). Some variation was observed between the different experiments, probably related to the experimental conditions, the different developmental stages, or the use of different cultivars. However, no expression of *TaMAX1a2-3D*, which also belongs to the MAX1a2 subgroup, was observed in the roots. *TaMAX1a1-4B*, *-4D*, and *TaMAX1d* homoeologues were also found to be expressed in the roots of some cultivars under N-sufficient conditions. In N-limited plants, due to changes in the expression of *TaMAX1* genes, the total transcript abundance of *TaMAX1a2* homoeologues accounted on average for 80% of the total transcript abundance of *TaMAX1* genes, indicating that under N-limiting conditions, the *TaMAX1a2* homoeologues are responsible for the biosynthesis of SLs in roots. No expression of *TaMAX1b-3A* and low expression of *TaMAX1c* homoeologues was detected in roots in either high or low N conditions in any of the cultivars, indicating that those genes do not function in roots.

In contrast, *TaMAX1c* homoeologues were the most abundant *MAX1* genes expressed in basal nodes, regardless of the N supply (Fig. 3B). In addition, *TaMAX1d* homoeologues also showed expression in basal nodes. *TaMAX1a2* homoeologues were not expressed in nodes in any of the samples included in the analysis (TPM <0.5). As observed in roots, *TaMAX1b-3A* was not expressed in basal nodes. In shoots, *TaMAX1c-6A* and *-6B* were the most abundantly expressed homoeologues. However, low expression of *TaMAX1d* was detected. In contrast, expression of *TaMAX1a1-4D* was detected in shoots and was higher than that of the A and B homoeologues (Fig. 3C).

### Response of SL-related genes to N limitation in wheat roots

**Fig. 4.**
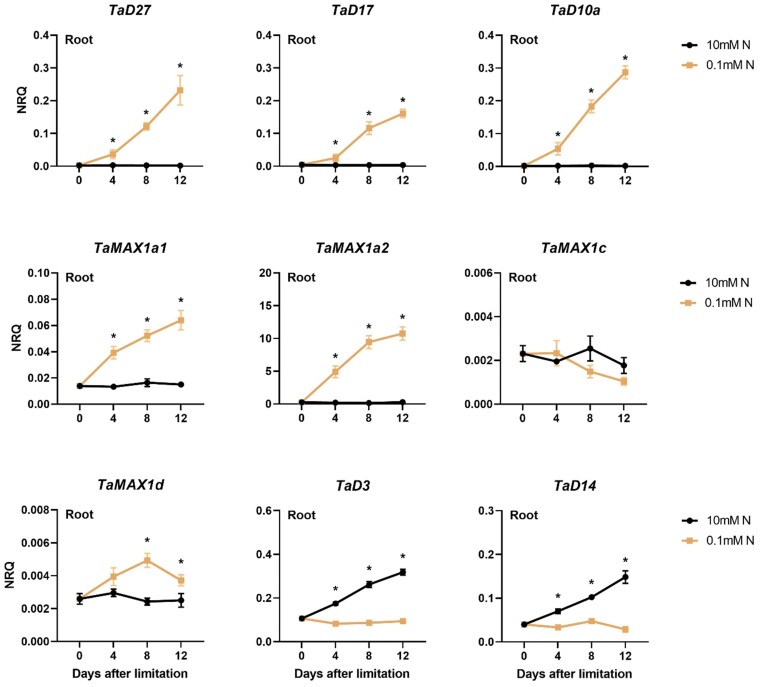
Time-course analysis of the gene expression levels of SL biosynthetic (*TaD27*, *TaD17*, *TaD10a*, *TaMAX1a1*, *TaMAX1a2*, *TaMAX1c*, and *TaMAX1d*) and perception genes (*TaD3* and *TaD14*) in the root of wheat (cv Cadenza) 0, 4, 8, and 12 d after N limitation. Plants were introduced to N limitation at 10 DAS. Values are means of three biological replicates, and error bars represent the SE. Statistical analysis was conducted with ANOVA on log_2_(1/NRQ) transformed data. * denotes a statistically significant difference in the gene expression levels between low N and high N plants at each time point based on Fisher’s LSD test.

To study the effect of N limitation on SL biosynthetic and perception genes, a time-course analysis of transcript levels in roots was conducted by RT–qPCR (Fig. 4; Supplementary Table S7). The expression of *TaD27*, *TaD17*, and *TaD10a* sharply increased over time in N-limited plants, whilst in high N-treated plants, the expression of those genes remained constant at low levels.

In roots, *TaMAX1a1*, *TaMAX1a2*, and *TaMAX1d* were induced by N limitation. *TaMAX1a2* showed the most substantial up-regulation by N limitation. In fact, *TaMAX1a2* transcript levels were found to be 60-fold higher in low N roots 8 d after N limitation. In high N plants, the expression of those genes remained stable at low levels at all the three time points examined, as also shown for the SL biosynthetic genes of the core pathway (*TaD27*, *TaD17*, and *TaD10a*). However, *TaMAX1c* was not found to be affected by N treatment in roots. This different response of *TaMAX1c* compared with the other *TaMAX1* genes indicates that there are some differences in the regulation of *MAX1* gene expression in response to N limitation.

The response of SL perception genes was examined in roots. In contrast to SL biosynthetic genes, the transcript abundances of *TaD3* and *TaD14* were significantly lower in the root of plants grown at low N availability, indicative of SL-mediated feedback regulation (Fig. 4; Supplementary Table S7). Under high N conditions, mRNA levels of *TaD3* and *TaD14* increased over time, whereas this was not observed in the roots of N-limited plants.

### Response of SL-related genes to N limitation in wheat basal nodes

**Fig. 5.**
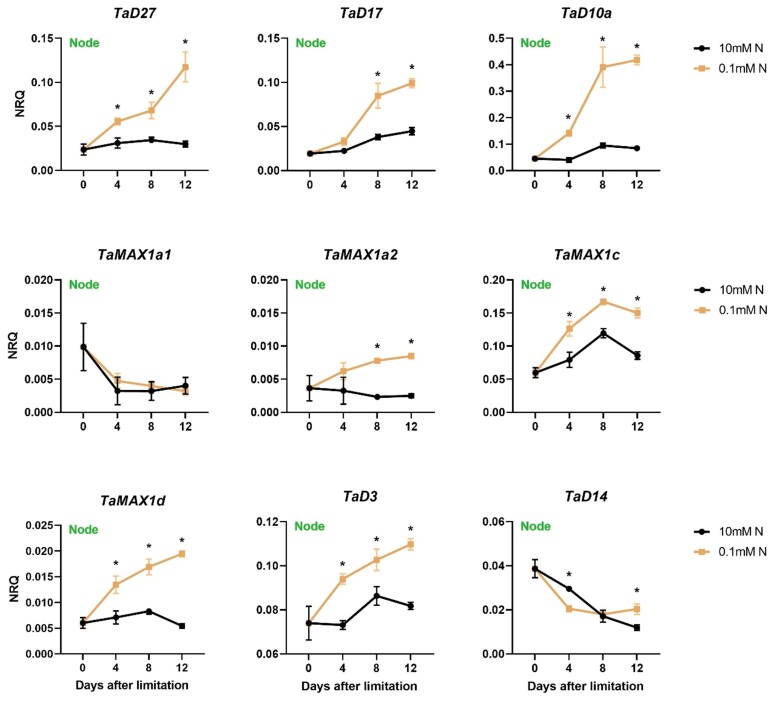
Time-course analysis of the gene expression levels of SL biosynthetic (*TaD27*, *TaD17*, *TaD10a*, *TaMAX1a1*, *TaMAX1a2*, *TaMAX1c*, and *TaMAX1d*) and perception genes (*TaD3* and *TaD14*) in the basal nodes of wheat (cv Cadenza) 0, 4, 8, and 12 d after N limitation. Plants were introduced to N limitation at 10 DAS. Values are means of three biological replicates and error bars represent the SE. Statistical analysis was conducted with ANOVA in log_2_(1/NRQ) transformed values. * denotes a statistically significant difference in the gene expression levels between low N and high N plants at each time point based on Fisher’s LSD test.

Low N supply significantly reduced the number of outgrown tillers in the time course analysis (Supplementary Fig. S8A, D, E). The effect was apparent 8 d after the N limitation, while the effect became stronger with continuing N limitation and, 12 d after the N limitation, the number of tillers in the low N-treated plants was 2-fold lower than that in the high N-treated plants. Therefore, the expression of SL biosynthetic and perception genes was examined in the basal node of wheat plants (Fig. 5; Supplementary Table S7). In this study, the basal node is defined as the 0.5 cm of the main shoot base, which includes the apical meristem, axillary buds, leaf meristems, etc.

As observed in roots, the transcript levels of *TaD27*, *TaD17*, and *TaD10a* in the basal node were gradually up-regulated in response to N limitation. More specifically, *TaD27* and *TaD10a* were found to be significantly induced at all the time points examined. However, *TaD17* was found to be significantly up-regulated from day 8 after the N limitation. *TaD10a* showed the most substantial up-regulation at all time points. *TaMAX1c* and *TaMAX1d* were found to be induced by N limitation from day 4 after N limitation onwards. No significant effect of N limitation was found in the expression of *TaMAX1a1*. ­However, the transcript abundance of *TaMAX1a2* increased in response to N limitation and was significantly induced on days 8 and 12 after N limitation.

Examination of the expression of genes involved in SL perception revealed that *TaD3* was slightly induced by N limitation in nodes. When compared with high N treatment, *TaD14* was down-regulated in the N-limited plants 4 d after N limitation. No difference was observed at 8 d after N limitation, while the mRNA levels were found to increase at 12 d after limitation. In the basal nodes, the expression of *TaTB1* was also examined (Supplementary Fig. S8C; Supplementary Table S5). The mRNA accumulation of *TaTB1* was significantly higher following 8 d and 12 d of N limitation. The induced expression of *TaTB1* correlates with the observed phenotype, given that the effect of N limitation on tiller formation and bud outgrowth became more apparent from day 8 after N limitation. This observation suggests that TB1 may be involved in tiller bud outgrowth suppression by N limitation in wheat.

### Comparative analysis of SL response to N and P limitation in basal nodes

**Fig. 6.**
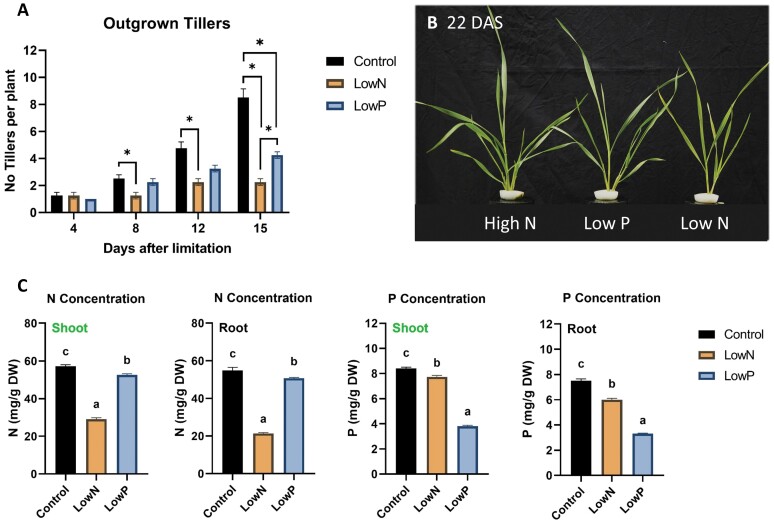
Effect of N and P limitation on wheat (cv Cadenza) tillering. (A) The number of outgrown tillers per plant at 4, 8, 12, and 15 d after N or P limitations. Plants were introduced to the respective nutrient limitation at 10 DAS. Values are means of four biological replicates, and error bars represent the SE. Statistical analysis was conducted with repeated measures ANOVA [LSD (5%)=0.998]. * denotes a statistically significant difference between the treatments at each time point based on Fisher’s LSD test. (B) Representative plants grown under control (10 mM N and 1 mM P), low P (10 mM N and 0.01 mM P), and low N (0.1 mM N and 1 mM P) conditions at 12 d (22 DAS) after the introduction of the plants to the nutrient limitation. (C) N and P concentration in the root and shoot of low N- and low P-treated plants. Values are means of four biological replicates, and error bars represent the SE. Different letters denote statistically significant differences between treatment means based on Fisher’s LSD test.

Both N and P limitations suppressed tiller numbers in wheat (Fig. 6A, B). However, the results suggested that N limitation had a more prominent effect on suppressing tillering than P limitation. N limitation strongly reduced the N concentration in both roots and shoots (Fig. 6C; Supplementary Table S11). The reduction in N concentration was more substantial in roots than in shoots, with the N concentration reduced by 60% in roots and by 50% in shoots. A statistically significant decrease in the N concentration was also observed in low P plants, but the reduction was only by 7–8% in both tissues examined. In relation to P content, a significant decrease in P concentration was observed in the root and shoot of low P-treated plants (Fig. 6C; Supplementary Table S11). However, a decline in the P concentration was also found in low N-treated plants. The effect of N limitation on P concentration was more prominent in roots, where P levels declined by 20%, while in shoots, P concentration was 8% lower in low N plants compared with the control. Although a decrease in P concentration was observed in low N plants, low N plants still showed twice higher P levels than the low P-treated plants.

**Fig. 7.**
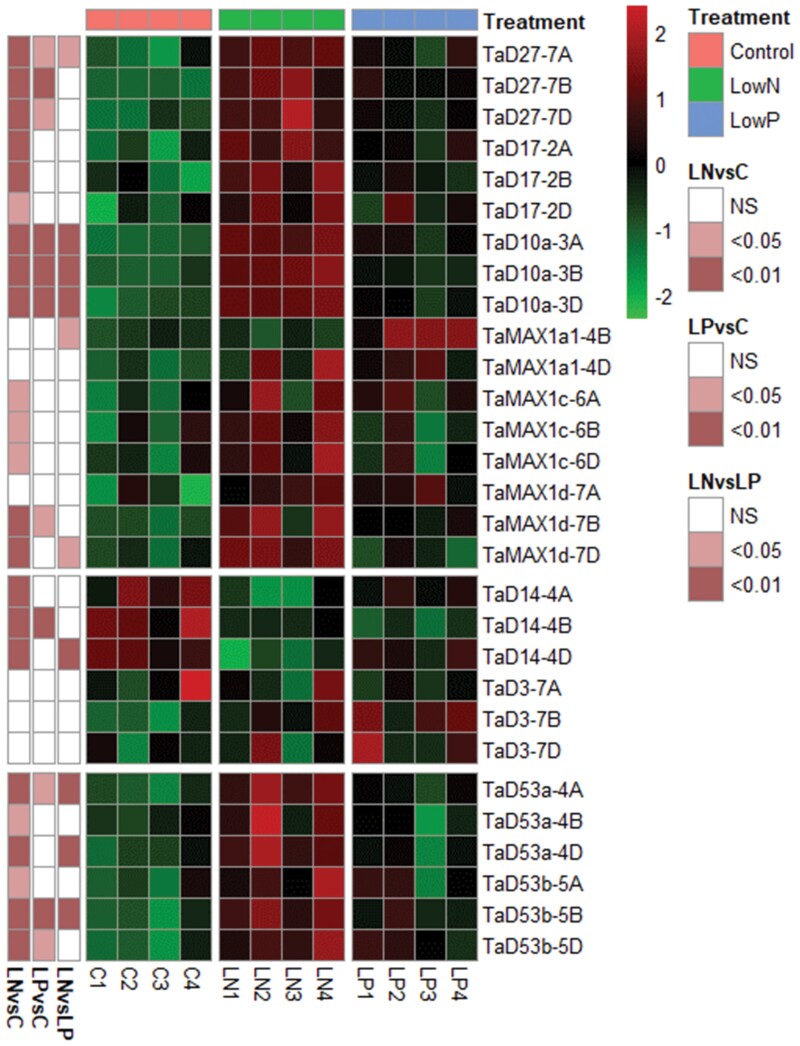
Heatmap comparison of SL biosynthetic, perception, and signalling gene transcript levels in the basal nodes of wheat (cv Cadenza) grown under N and P limitation for 8 d based on the RNA-seq data. Each row corresponds to a different gene, while columns correspond to different samples grouped by treatment. Only SL-related genes that were found to be expressed (TPM >0.5) in the basal node were included in the heatmap. Data are *Z*-scores of regularized log-normalized counts (rlog) as generated by the DESeq2 tool. Red colour corresponds to higher transcript levels while green corresponds to lower transcript levels. Row annotations represent the *P*adj values of each comparison according to the differential gene expression analysis (Supplementarty Table S8).

P supply is considered the primary signal controlling SL biosynthesis, and SLs are responsible for tillering regulation by P availability (Umehara *et al.*, 2010; Kohlen *et al.*, 2011; Yoneyama *et al.*, 2012). Therefore, RNA-seq was performed for the comparative analysis of the transcriptional effect of N and P limitation on SL biosynthetic, perception, and signalling in the basal nodes (Fig. 7; Supplementary Fig. S9; Supplementary Tables S8, S9). N limitation strongly induced the expression of *TaD27*, *TaD17*, and *TaD10a* homoeologues. *TaD10a* showed the highest FC increase, >4-fold in N-limited basal nodes. Similarly, significant overexpression was observed for *TaMAX1d-7B* and -*7D*. Other *MAX1d* and *MAX1c* homoeologues showed higher transcript accumulation; however, the FC difference or *P*adj values did not meet the applied thresholds. Finally, mRNA accumulation of *TaD53a-4A*, *-4D*, *TaD53b-5B*, and *-5D* increased under N limitation in the basal nodes. Analysis of the differentially expressed genes in P-limited plants showed that only *TaD27-7B* and three homoeologues of *TaD10a* were up-regulated in nodes. In addition, the recorded up-regulation of *TaD10*a homoeologues by P limitation was 2-fold higher, while the same genes were 5-fold higher in N-limited nodes. As a result, it is suggested that N limitation had a more substantial effect on SL biosynthesis and signalling genes compared with P limitation in the nodes.

To further confirm this observation, differential gene expression analysis was performed between low N and low P plants to identify genes with significant differences between the two treatments. The differential gene expression analysis results confirmed that the mRNA abundance of some SL biosynthetic genes, such as *TaD10a*, was significantly higher (*P*adj <0.01 and FC >1.5) in low N nodes compared with low P (Fig. 7; Supplementary Table S8). Statistically significant ­differences were observed between low N and low P nodes in the case of *TaD53a-4A*, *-4D*, and *TaD53b-5B*, indicating that under N limitation, the levels of SLs were presumably higher in this tissue, given that *TaD53* expression is regulated by the levels of SLs.

### Comparative analysis of SL response to N and P limitation in roots

**Fig. 8.**
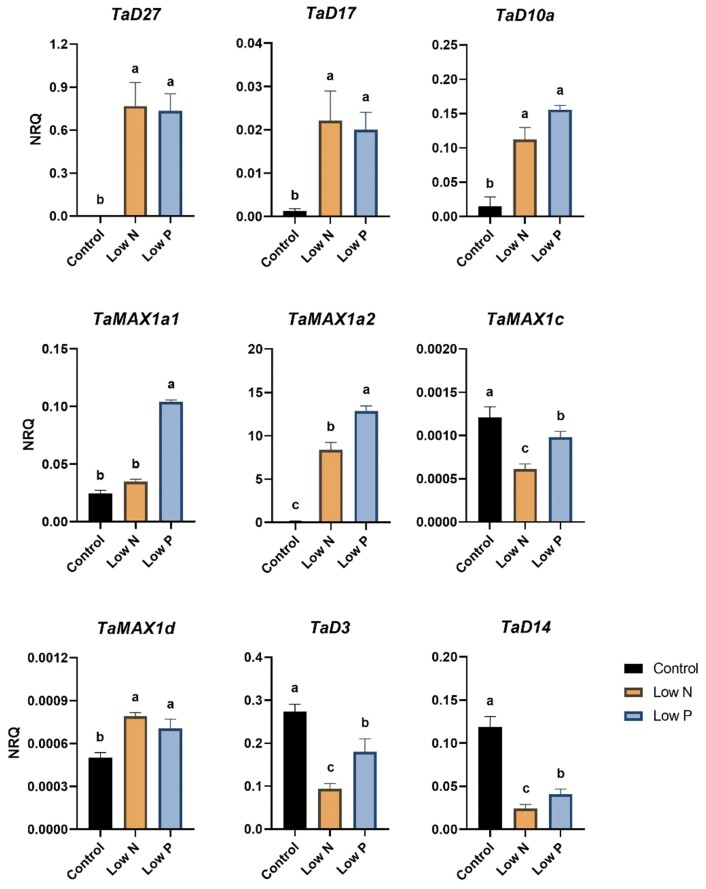
Gene expression levels of SL biosynthetic (*TaD27*, *TaD17*, *TaD10a*, *TaMAX1a1*, *TaMAX1a2*, *TaMAX1c*, and *TaMAX1d*) and perception genes (*TaD3* and *TaD14*) in the root of wheat (cv Cadenza) grown under N and P limitation for 8 d based on RT–qPCR data. Values are means of four biological replicates and error bars represent the SE. Statistical analysis was conducted with ANOVA in log_2_(1/NRQ) transformed values. Different letters denote statistically significant differences between treatment means based on Fisher’s LSD test.

The findings in the basal nodes showed that N limitation led to stronger induction of SL biosynthesis compared with P ­limitation. The same comparison was conducted in the root, where the gene expression of SL biosynthesis and perception genes was performed by RT–qPCR (Fig. 8; Supplementary Table S10). Overall, the results demonstrated that SL biosynthetic genes were strongly induced by both nutrient limitations, while the examined SL perception genes showed a significant down-regulation.

Although N limitation led to stronger induction of SL biosynthesis compared with P limitation in the basal nodes, the mRNA accumulation of SL biosynthetic genes in roots was as high or even higher in low P roots compared with low N roots. No significant difference was observed between the mRNA accumulation of *TaD27* and *TaD17* between low N and low P treatments. A stronger expression of *TaD10* was recorded under low P conditions. However, no significant difference was observed between the two nutrient limitations. The main differences in the effect of N and P limitation on SL biosynthesis were observed in *TaMAX1* gene expression. In fact, P-limited plants showed significantly higher mRNA levels of *TaMAX1a1* and *TaMAX1a2* in roots compared with low N plants. In terms of SL perception, both conditions strongly down-regulated the expression of *TaD3* and *TaD14* in roots. In fact, TaD3 and TaD14 were found to be significantly lower in low N roots compared with low P roots.

## Discussion

### SL biosynthesis and signalling genes are regulated by N availability

N is an essential macronutrient for plant growth. Plant adaptation to N-limiting conditions includes changes in root and shoot architecture in such a way as to increase the use efficiency of available resources and maximze the chances of survival and successful reproductive development. The results presented in this study demonstrated that shoot architecture is strongly affected by N availability. N limitation strongly suppressed tiller outgrowth, resulting in a reduced number of tillers per plant. As plant N levels decreased, the effect of N limitation on tillering became more apparent by affecting the formation of higher order tillers (Supplementary Fig. S8). In Arabidopsis, branching is suppressed by plant N status and not by the NO_3_^–^ content of shoots, as different sources of N had the same effect on branching. This suggests that tiller suppression is controlled by systemic signals rather than by the local concentration of NO_3_^–^ (de Jong *et al.*, 2014).

In many species, N limitation has been found to promote SL biosynthesis and exudation. This response has also been observed in wheat under N or P limitation (Yoneyama *et al.*, 2012). However, few studies have focused on the transcriptional regulation of SL biosynthetic and signalling genes by N availability. To investigate the interaction between N and SLs in wheat, the expression levels of the major biosynthetic, perception, and signalling genes were monitored in roots and basal nodes of wheat grown under high and low N supply. The results showed that N limitation strongly induces the expression of the biosynthetic genes examined. Thus, although changes in gene expression do not necessarily correlate with protein abundance, it may be that the previously reported induction of SL levels under N-limiting conditions in wheat is due to the strong up-regulation of SL biosynthetic genes (Yoneyama *et al.*, 2012). Similar transcriptional regulation of *D27* and *D17* has been reported in rice roots upon N limitation (Sun *et al.*, 2014). Moreover, the mRNA levels of SL biosynthetic genes were found to be down-regulated in roots after transferring N-limited plants to high N conditions (Supplementary Fig. S10). These findings support the conclusion that SL biosynthetic genes are tightly regulated by plant N supply.

SL perception genes, *TaD3* and *TaD14*, were found to be strongly down-regulated in the roots of low N plants. D3 and D14 expression has been reported to be regulated by a negative feedback loop (Umehara *et al.*, 2010; Sun *et al.*, 2014; Marzec and Melzer, 2018). Therefore, this can explain the observed down-regulation in low N roots and further support that N limitation leads to elevated levels of SLs by up-regulating the SL biosynthetic genes. However, the same response was not observed in basal nodes.

SLs are thought to be predominantly produced in roots. However, the expression of biosynthetic genes has also been detected in axillary buds. Grafting experiments in dicotyledonous species have demonstrated that locally produced SLs in shoots are sufficient to control shoot branching (Sorefan *et al.*, 2003; Booker *et al.*, 2004). According to the current results, expression of SL biosynthetic genes was detected in basal nodes of wheat plants, where N limitation induced their expression, as was also observed in roots. Although *TaMAX1c* did not show any response to N level in roots, it was significantly induced in basal nodes by N limitation. The opposite was true for *MAX1a1* genes, suggesting a tissue-specific regulation of those genes (further discussed below). Expression of *TaD53* homoeologues was found to be induced by N limitation, probably due to the degradation of D53 and the transcriptional release of *TaD53* in the presence of higher SL levels. This observation further supports that N limitation leads to an increase in SL levels locally in the basal nodes. Similarly, *TaTB1*, which is considered a downstream target of the SL signalling pathway, was also induced by N limitation. It is well established that SLs are involved in repressing tiller bud outgrowth under P limitation in rice and Arabidopsis (Umehara *et al.*, 2010; Kohlen *et al.*, 2011). P limitation also induces SL biosynthesis locally in rice buds. As a result, the recorded induction of SL biosynthetic genes and, therefore, SL biosynthesis in basal nodes might contribute to tiller inhibition under N-limiting conditions. J. Wang *et al.* (2018) also showed a link between N uptake and SL biosynthesis. More specifically, rice *NPF7.2*-overexpressing lines showed enhanced tillering and NO_3_^–^ uptake, whilst the expression of SL biosynthetic genes was strongly down-regulated in tiller buds. The opposite effect was observed for *npf7.2* mutant lines (J. Wang *et al.*, 2018). Moreover, SL biosynthetic genes (*D27*, *D17*, and *D10*) were suppressed in the tiller nodes within 3 h of N supply (Xu *et al.*, 2015). Consequently, local SL biosynthesis in nodes is regulated by N availability and possibly participates in the tiller regulation. However, de Jong *et al.* (2014) showed that N-mediated bud outgrowth regulation is at least partly dependent on SLs in Arabidopsis. SL mutants were found to form more branches than wild-type plants under low N conditions, but SL mutants could still respond to N availability. Similar results were later reported for rice *d3* and *d10* mutants (Luo *et al.*, 2018).

### Different nutrient signals regulate SL biosynthesis in below- and above-ground tissue

The results showed that N limitation strongly induced SL biosynthesis in the root and the basal nodes. In rice, it has been demonstrated that tiller suppression by P limitation is mediated by SLs (Umehara *et al.*, 2010). SL biosynthesis is strongly induced in rice root and shoot bases of plants grown under P limitation, leading to tiller suppression. In contrast, SL induction by N limitation has been speculated to be due to the reduced P uptake by N-deprived plants, rather than the direct effect of N limitation (Yoneyama *et al.*, 2012). However, those studies have mainly referred to SL levels in root exudates or in root tissue where P limitation has a predominant impact on SL production. However, less is known about the transcriptional regulation of SL-related genes in the upper part of the plants. Based on the RNA-seq data in the basal nodes, the N limitation led to a more substantial up-regulation of many SL biosynthetic genes compared with P limitation. Differential gene expression analysis between low N and low P plants showed that the transcript levels of many SL biosynthetic and signalling genes were significantly different between the two treatments. Assuming that shoot P status is the primary signal controlling SL biosynthesis in nodes, one explanation could be that the internal concentration of P was not so low as to trigger a low P response. However, the elemental analysis showed that low P plants had a 2-fold lower concentration of P in shoots. N-limited plants showed reduced P content in shoots, but the concentration was much higher than in low P plants. As a result, the P content in the shoot cannot explain the strong up-regulation of SL biosynthetic genes in the basal nodes.

However, gene expression analysis in roots by RT–qPCR revealed that P limitation had a more substantial effect on SL biosynthesis genes than N limitation. Therefore, the results presented suggested that P limitation triggered a more potent induction of SL biosynthesis compared with N limitation in the root, especially in the case of *TaMAX1* genes of clade A. Yoneyama *et al.* (2012) had indeed reported that SL exudation is stronger in wheat plants growing under P-limiting conditions compared with low N, which is also supported by the transcriptomic data.

Combining these results, it is suggested that SL biosynthesis is regulated by different signals in roots and shoots in response to plant nutritional status. SLs are known to have a dual role as a rhizosphere signal and as plant phytohormones. In roots, production and exudation of SLs have been associated with AMF colonization, which facilitates P uptake under P deficiency. However, in nodes, SLs have a role as a negative regulator of bud outgrowth. Consequently, we can hypothesize that in roots, P limitation strongly induced SL biosynthesis to increase SL exudation to facilitate AMF colonization and increase P capture, while in nodes SLs act mainly as phytohormone coordinating bud suppression. Wheat tillering was found to be more sensitive to changes in N availability compared with P (Fig. 6). As a result, the more potent induction of SL biosynthesis correlates well with the phenotypic response.

Based on the analysis of P content performed in this study, N limitation negatively impacted P content in both roots and shoots, but the concentrations found in N-limited plants were significantly higher compared with those observed in low P plants. Combining these two observations, the P content is not the signal controlling SL biosynthesis in nodes, as the induction of SL biosynthesis was weaker under P limitation. Therefore, there must be other factors which regulate SL biosynthesis, at least locally in the nodes. In split-root experiments in sorghum, it was found that N or P themselves were not the signals controlling SL biosynthesis gene expression in roots (Yoneyama *et al.*, 2015, 2020b). In addition, in the same studies, it was suggested that neither auxin nor cytokinins (CKs) were responsible for controlling SL biosynthesis in response to nutritional signals, although both hormones affected the expression of the genes when applied externally. In the present study, the levels of most of the CKs were strongly down-regulated in N-limited roots, while no effect was observed under P limitation (Supplementary Fig. S11). Therefore, CKs cannot be the signal leading to strong up-regulation of SL biosynthetic genes in roots under P-limiting conditions.

### Wheat *MAX1* homologues show distinct expression patterns and potentially distinct functions

In Arabidopsis, there is only one *MAX1* gene that catalyses the conversion of CL to CLA, which is subsequently used as a substrate by a recently identified SABATH-methyltransferase and which produces Me-CLA (Wakabayashi *et al.*, 2021). Me-CLA is further metabolized by LBO for the synthesis of 1ʹ-OH-MeCLA (Brewer *et al.*, 2016; Yoneyama *et al.*, 2020a). Both Me-CLA and 1ʹ-OH-MeCLA have shown activity as branching inhibitors, indicating that non-canonical SL might be responsible for the branching inhibition activity of SLs (Abe *et al.*, 2014; Brewer *et al.*, 2016). However, five *MAX1* homologues have been identified in the rice genome (Zhang *et al.*, 2014), while more recent studies showed that the presence of multiple *MAX1* genes is a common characteristic in grasses. More specifically, maize has three *MAX1* genes (Yoneyama *et al.*, 2018), sorghum has four (Wu and Li, 2021), and *B. distachyon* has five (Changenet *et al.*, 2021). According to some studies, MAX1s from grasses fall into four different clades (Marzec *et al.*, 2020; Wu and Li, 2021), whereas Vinde *et al.* (2022) only accounted for the presence of three separate groups. The multiple MAX1 homologues have been suggested to catalyse different steps in the conversion of CL to bioactive SL molecules. Although progress has been made in understanding the function of *MAX1* genes, especially in rice, their specific function remains elusive. Wheat was found to have 13 distinct *MAX1* genes in its genome. Phylogenetic analysis also showed that MAX1s from grasses form four different clades. The results here are consistent with Marzec *et al.* (2020), who reported the presence of four distinct clades, but in their study only eight out of the 13 identified wheat MAX1 sequences had been included (Marzec *et al.*, 2020). In clade B, only a single wheat MAX1 was found along with three rice orthologues and one representative from all the other grasses included in the study. Interestingly, two different homoeologous subgroups were found in clade A, while only *B. distachyon* was found to have closely related sequences to both subgroups of clade A. No rice orthologue was present in clade A.

Recent studies have shown that both Os900 and Os1400 convert CL to CLA, whilst each one has an additional specific activity (Yoneyama *et al.*, 2018). However, Os1500 does not show enzymatic activity, which has been attributed to a premature stop codon. More specifically, Os900 converts CLA to 4DO, and Os1400 converts 4DO to orobanchol (Zhang *et al.*, 2014). Orobanchol is a canonical SL which is mainly found in root exudates, indicating that it acts as a rhizosphere signal. Similarly, Yoneyama *et al.* (2018) also showed that ZmMAX1b, which belongs to the same clade (clade B), also has similar activity to Os1400. According to Umehara *et al.* (2010), the expression of Os1400 was only found in rice roots, while mRNA was undetected in lateral buds (Umehara *et al.*, 2010). Based on the current RNA-seq data, *TaMAX1b-3A*, which is closely related to Os1400, Os900, and Os5100, was not expressed in any of the tissues examined. In comparison, members of the *TaMAX1a2* subgroup were expressed exclusively in roots, and their expression was strongly induced by N and P limitations. A similar, root-specific expression of *BdCYP711A31*, which also belongs to clade A2, has been reported in *B. distachyon* (Changenet *et al.*, 2021). Therefore, based on the expression patterns, it is assumed that members of subgroup A2 have a similar function to Os1400 and Os900, and are therefore involved in the biosynthesis of canonical SLs such as 4DO or orobanchol in roots. In wheat and other species, N and P limitation stimulates the biosynthesis of orobanchol in roots (Yoneyama *et al.*, 2012). *TaMAX1a2-3B* and *TaMAX1a2-U* showed a significant induction and were the predominant *MAX1* genes expressed in roots under low N conditions. The same response was consistent for different experiments and cultivars. Thus, this observation indicates that members of clade A play predominant roles in SL biosynthesis in roots. Furthermore, SbMAX1a, which is phylogenetically close to TaMAX1a2, has been shown to be involved in the production of orobanchol in sorghum, further supporting this hypothesis (Wu and Li, 2021).

Os1900 and Os5100, which belong to clades C and D, respectively, have shown weak activity for conversion of CL to CLA based on yeast microsome studies (Yoneyama *et al.*, 2018). In the same study, Yoneyama *et al.* (2018) showed that ZmMAX1c and ZmMAX1a, which are phylogenetically close to Os1900 and Os5100, respectively, had weak activity for conversion of CL to CLA, indicating that CL is not the preferred substrate of MAX1 enzymes belonging to those clades. The gene expression data in the present study revealed that *TaMAX1c* homoeologues are orthologues of Os1900 and are only expressed in nodes. More specifically, *TaMAX1c* genes were the predominant *MAX1* genes expressed in nodes and shoots, while no expression was detected in roots. This observation can explain why *TaMAX1c* mRNA levels were found to be up-regulated by N limitation only in nodes and not in roots, based on the N limitation time-course gene expression analysis. This finding is consistent with expression analysis in *B. distachyon*, in which transcript levels of *BdCYP711A5* were higher in leaves compared with roots (Changenet *et al.*, 2021). SbMAX1c, which is closely related to TaMAX1c homoeologues, in addition to catalysing the conversion of CL to CLA, also catalyses the production of an unknown peak, indicating the production of an as yet unidentified SL-like compound with a similar molecular weight to 18-hydroxy-CLA (Wu and Li, 2021). Marzec *et al.* (2020), based on *in silico* analysis, speculated that Os1900 might be involved in the biosynthesis of non-canonical rather than canonical SL. The present expression analysis supports this hypothesis that TaMAX1cs might be involved in the production of unknown SLs or SL intermediates, which are produced and function in above-ground tissues acting in the regulation of shoot architecture. Expression of *TaMAX1d* homoeologues was found in both tissues, suggesting that MAX1d function is required in both the root and shoots. However, the expression levels (TPM) were higher in nodes compared with roots.

## Conclusion

This study demonstrates that SL biosynthetic genes are strongly affected by N limitation in both root and basal nodes of wheat. Based on the RNA-seq experiment, it was shown that in the basal nodes, N limitation leads to stronger induction than P limitation, whilst the opposite response was observed in roots, indicating a tissue-specific regulation of SL biosynthetic genes by nutritional signals. Finally, a phylogenetic analysis of MAX1 present in wheat was performed along with a systematic analysis of the MAX1-encoding gene expression patterns. In conclusion, the results showed a clear tissue-specific expression and regulation of *TaMAX1* homologues in response to N limitation, suggesting different functionalities and roles of MAX1 in wheat. Further studies manipulating specific *MAX1* genes are required in order to better understand the functional diversity and their roles in wheat, and also to understand the structural diversity of SLs produced in wheat.

## Supplementary data

The following supplementary data are available at *JXB* online.

Table S1. Gene IDs of the identified SL-related genes in wheat (IWGSC RefSeq Annotation v1.1) and gene IDs from other species used for the identification of wheat genes.

Table S2. Primer sequence used in the RT–qPCR expression analysis.

Table S3. List of RNA-seq samples used for the expression analysis of *TaMAX1* homologues.

Table S4. Protein sequence percentage identity matrices of the identified SL-related wheat genes and of other species used for the identification of wheat genes.

Table S5. Gene IDs of CYP711A/MAX1 from different species used for the phylogenetic analysis.

Table S6. Mean transcript abundance (TPM) of the *TaMAX1* homologues based on different RNA-seq data.

Table S7. Normalized relative quantity (NRQ) data of the RT–qPCR expression analysis in the root and basal nodes of wheat from the N limitation time-course analysis.

Table S8. Differential gene expression analysis results for SL-related genes in the basal nodes of N- and P-limited plants.

Table S9. TPM values of SL-related genes in the basal nodes of N- and P-limited plants.

Table S10. Normalized relative quantity (NRQ) data of the RT–qPCR expression analysis in the root of N- and P-limited plants.

Table S11. Elemental analysis results in N- and P-limited plants (mg g DW^–1^).

Fig. S1. RNA-seq validation test results.

Fig. S2. Phylogenetic relationship and protein alignment of D17/CCD7 and D10/CCD8 proteins from hexaploid wheat, rice, barley, and Arabidopsis.

Fig. S3. Heatmap of putative *TaD10* expression profiles in different tissues and under different conditions extracted from expVIP Wheat expression Browser.

Fig. S4. Protein alignment and phylogenetic relationship of D53 proteins from hexaploid wheat, rice, sorghum, maize, and Arabidopsis.

Fig. S5. Protein alignment and phylogenetic relationship of CYP711A/MAX1 proteins mainly from grasses such as hexaploid wheat, rice, barley, sorghum, maize, and *B. distachyon*, from dicotyledons such as Arabidopsis, *Solanum lycopersicum*, *Vitis vinifera*, and *Amborella trichopoda*, and from the lycophyte *Selaginella moellendorffii*.

Fig. S6. Nucleotide sequence alignment of hexaploid wheat and *T. dicoccoides MAX1a2-3B* genomic sequences.

Fig. S7. Heatmap comparison of *TaMAX1* gene expression values expressed as TPM in root and shoot of P-sufficient and P-stressed wheat plants.

Fig. S8. Time-course analysis of the effect of N limitation on tillering, root N concentration, and gene expression levels of *TaTB1* in the basal nodes of wheat (cv Cadenza) 0, 4, 8, and 12 d after N limitation. Representative wheat plants (cv Cadenza) grown under high N and low N conditions at 8 d (18 DAS) and 12 d (22 DAS) after N limitation.

Fig. S9. Transcript abundance of SL biosynthetic (*TaD27*, *TaD17*, *TaD10a*, *TaMAX1a1*, *TaMAX1a2*, *TaMAX1c*, and *TaMAX1d*), perception (*TaD3* and *TaD14*) and signalling (*TaD53a* and *TaD53b*) genes and their homoeologues in the basal nodes of wheat (cv Cadenza) grown under N and P limitation for 8 d based on the RNA-seq data.

Fig. S10. Time-course analysis of the gene expression levels of SL biosynthetic (*TaD27*, *TaD17*, *TaD10a*, *TaMAX1a1*, and *TaMAX1a2*) and perception genes (*TaD3* and *TaD14*) in the root of wheat (cv Cadenza) before (0 h), 24 h, and 72 h after N resupply to N-limited plants.

Fig. S11. Concentration of different CKs (pg mg^–1^) in the root of wheat (cv Cadenza) grown under control (10 mM N and 1 mM P), low N (0.1 mM N), and low P (0.01 mM P) conditions for 8 d.

erad008_suppl_Supplementary_Figures_S1-S11

erad008_suppl_Supplementary_Tables_S1-S11

## Data Availability

All data supporting the findings of this study are available within the paper and within its supplementary data published online. The raw data from RNA-seq are available at the ArrayExpress (E-MTAB-11986).

## Abbreviations

AMF: arbuscular mycorrhizal fungi
CCD: CAROTENOID CLEAVAGE DIOXYGENASE
CK: cytokinin
CL: carlactone
CLA: carlactonoic acid
DAS: days after sowing
4DO: 4-deoxyorobanchol
FC: fold change
LBO: LATERAL BRANCHING OXIDOREDUCTASE
MAX: MORE AXILLARY GROWTH
N: nitrogen
NGS: next-generation sequencing
NRQ: normalized relative quantity
P: phosphorus
RT–qPCR: quantitative reverse transcription–PCR
SCF: Skp1–Cullin–F-box
SL: strigolactone
SMXL: SUPPRESSOR OF MAX2 1-LIKE
TB1: TEOSINTE BRANCHED 1
TPM: transcripts per million mapped reads

## References

[CIT0001] Abe S , Sado A, Tanaka K, et al. 2014. Carlactone is converted to carlactonoic acid by MAX1 in Arabidopsis and its methyl ester can directly interact with AtD14 *in vitro*. Proceedings of the National Academy of Sciences, USA 111, 18084–18089. 10.1073/pnas.1410801111 PMC4273391 25425668

[CIT0002] Aguilar-Martínez JA , Poza-Carrión C, Cubas P. 2007. Arabidopsis BRANCHED1 acts as an integrator of branching signals within axillary buds. The Plant Cell 19, 458–472. 17307924 10.1105/tpc.106.048934 PMC1867329

[CIT0003] Akiyama K , Matsuzaki K-I, Hayashi H. 2005. Plant sesquiterpenes induce hyphal branching in arbuscular mycorrhizal fungi. Nature 435, 824–827. 10.1038/nature03608 15944706

[CIT0004] Alder A , Jamil M, Marzorati M, Bruno M, Vermathen M, Bigler P, Ghisla S, Bouwmeester H, Beyer P, Al-Babili S. 2012. The path from β-carotene to carlactone, a strigolactone-like plant hormone. Science 335, 1348–1351. 22422982 10.1126/science.1218094

[CIT0005] Booker J , Auldridge M, Wills S, McCarty D, Klee H, Leyser O. 2004. MAX3/CCD7 is a carotenoid cleavage dioxygenase required for the synthesis of a novel plant signaling molecule. Current Biology 14, 1232–1238. 15268852 10.1016/j.cub.2004.06.061

[CIT0006] Bray NL , Pimentel H, Melsted P, Pachter L. 2016. Near-optimal probabilistic RNA-seq quantification. Nature Biotechnology 34, 525–527. 10.1038/nbt.3519 27043002

[CIT0007] Brewer PB , Yoneyama K, Filardo F, et al. 2016. LATERAL BRANCHING OXIDOREDUCTASE acts in the final stages of strigolactone biosynthesis in Arabidopsis. Proceedings of the National Academy of Sciences, USA 113, 6301–6306. 10.1073/pnas.1601729113 PMC4896730 27194725

[CIT0008] Changenet V , Macadré C, Boutet-Mercey S, Magne K, Januario M, Dalmais M, Bendahmane A, Mouille G, Dufresne M. 2021. Overexpression of a cytochrome P450 monooxygenase involved in orobanchol biosynthesis increases susceptibility to fusarium head blight. Frontiers in Plant Science 12, 662025. 33868356 10.3389/fpls.2021.662025 PMC8048717

[CIT0009] Chesterfield RJ , Vickers CE, Beveridge CA. 2020. Translation of strigolactones from plant hormone to agriculture: achievements, future perspectives, and challenges. Trends in Plant Science 25, 1087–1106. 32660772 10.1016/j.tplants.2020.06.005

[CIT0010] Cook CE , Whichard LP, Turner B, Wall ME, Egley GH. 1966. Germination of witchweed (*Striga lutea* Lour.): isolation and properties of a potent stimulant. Science 154, 1189–1190. 17780042 10.1126/science.154.3753.1189

[CIT0011] de Jong M , George G, Ongaro V, Williamson L, Willetts B, Ljung K, McCulloch H, Leyser O. 2014. Auxin and strigolactone signaling are required for modulation of arabidopsis shoot branching by nitrogen supply. Plant Physiology 166, 384–395. 25059707 10.1104/pp.114.242388 PMC4149722

[CIT0012] Drew MC , Saker LR. 1984. Uptake and long-distance transport of phosphate, potassium and chloride in relation to internal ion concentrations in barley: evidence of non-allosteric regulation. Planta 160, 500–507. 24258776 10.1007/BF00411137

[CIT0013] Edgar RC. 2004. MUSCLE: multiple sequence alignment with high accuracy and high throughput. Nucleic Acids Research 32, 1792–1797. 15034147 10.1093/nar/gkh340 PMC390337

[CIT0014] Gomez-Roldan V , Fermas S, Brewer PB, et al. 2008. Strigolactone inhibition of shoot branching. Nature 455, 189–194. 18690209 10.1038/nature07271

[CIT0015] Kersey PJ , Allen JE, Allot A, et al. 2018. Ensembl genomes 2018: an integrated omics infrastructure for non-vertebrate species. Nucleic Acids Research 46, D802–D808. 29092050 10.1093/nar/gkx1011 PMC5753204

[CIT0016] Kibbe WA. 2007. OligoCalc: an online oligonucleotide properties calculator. Nucleic Acids Research 35, W43–W46. 17452344 10.1093/nar/gkm234 PMC1933198

[CIT0017] Kobae Y , Kameoka H, Sugimura Y, Saito K, Ohtomo R, Fujiwara T, Kyozuka J. 2018. Strigolactone biosynthesis genes of rice are required for the punctual entry of arbuscular mycorrhizal fungi into the roots. Plant and Cell Physiology 59, 544–553. 29325120 10.1093/pcp/pcy001

[CIT0018] Kohlen W , Charnikhova T, Liu Q, Bours R, Domagalska MA, Beguerie S, Verstappen F, Leyser O, Bouwmeester H, Ruyter-Spira C. 2011. Strigolactones are transported through the xylem and play a key role in shoot architectural response to phosphate deficiency in nonarbuscular mycorrhizal host Arabidopsis. Plant Physiology 155, 974–987. 21119045 10.1104/pp.110.164640 PMC3032481

[CIT0019] Liu J , Cheng X, Liu P, Sun J. 2017. miR156-targeted SBP-box transcription factors interact with DWARF53 to regulate TEOSINTE BRANCHED1 and BARREN STALK1 expression in bread wheat. Plant Physiology 174, 1931–1948. 28526703 10.1104/pp.17.00445 PMC5490914

[CIT0020] Love MI , Huber W, Anders S. 2014. Moderated estimation of fold change and dispersion for RNA-seq data with DESeq2. Genome Biology 15, 550. 25516281 10.1186/s13059-014-0550-8 PMC4302049

[CIT0021] Luo L , Pan S, Liu X, Wang H, Xu G. 2017. Nitrogen deficiency inhibits cell division-determined elongation, but not initiation, of rice tiller buds. Israel Journal of Plant Sciences 64, 32–40.

[CIT0022] Luo L , Wang H, Liu X, et al. 2018. Strigolactones affect the translocation of nitrogen in rice. Plant Science 270, 190–197. 29576072 10.1016/j.plantsci.2018.02.020

[CIT0023] Luo L , Zhang Y, Xu G. 2020. How does nitrogen shape plant architecture? Journal of Experimental Botany 71, 4415–4427. 32279073 10.1093/jxb/eraa187 PMC7475096

[CIT0024] Martin M. 2011. Cutadapt removes adapter sequences from high-throughput sequencing reads. EMBnet.journal 17, 10–12.

[CIT0025] Marzec M , Melzer M. 2018. Regulation of root development and architecture by strigolactones under optimal and nutrient deficiency conditions. International Journal of Molecular Sciences 19, 1887. 29954078 10.3390/ijms19071887 PMC6073886

[CIT0026] Marzec M , Situmorang A, Brewer PB, Brąszewska A. 2020. Diverse roles of MAX1 homologues in rice. Genes 11, 1348. 33202900 10.3390/genes11111348 PMC7709044

[CIT0027] Mashiguchi K , Seto Y, Yamaguchi S. 2021. Strigolactone biosynthesis, transport and perception. The Plant Journal 105, 335–350. 33118266 10.1111/tpj.15059

[CIT0028] Matusova R , Rani K, Verstappen FW, Franssen MC, Beale MH, Bouwmeester HJ. 2005. The strigolactone germination stimulants of the plant-parasitic *Striga* and *Orobanche* spp. are derived from the carotenoid pathway. Plant Physiology 139, 920–934. 16183851 10.1104/pp.105.061382 PMC1256006

[CIT0029] Nei M , Saitou N. 1987. The Neighbor–Joining method: a new method for reconstructing phylogenetic trees. Molecular Biology and Evolution 4, 406–425. 3447015 10.1093/oxfordjournals.molbev.a040454

[CIT0030] Oono Y , Kobayashi F, Kawahara Y, Yazawa T, Handa H, Itoh T, Matsumoto T. 2013. Characterisation of the wheat (*Triticum aestivum* L.) transcriptome by *de novo* assembly for the discovery of phosphate starvation-responsive genes: gene expression in Pi-stressed wheat. BMC Genomics 14, 77. 23379779 10.1186/1471-2164-14-77 PMC3598684

[CIT0031] Ramakers C , Ruijter JM, Deprez RHL, Moorman AFM. 2003. Assumption-free analysis of quantitative real-time polymerase chain reaction (PCR) data. Neuroscience Letters 339, 62–66. 12618301 10.1016/s0304-3940(02)01423-4

[CIT0032] Ramírez-González RH , Borrill P, Lang D, et al. 2018. The transcriptional landscape of polyploid wheat. Science 361, 6403. 10.1126/science.aar6089 30115782

[CIT0033] Rieu I , Powers SJ. 2009. Real-time quantitative RT–PCR: design, calculations, and statistics. The Plant Cell 21, 1031–1033. 19395682 10.1105/tpc.109.066001 PMC2685626

[CIT0034] Shabek N , Ticchiarelli F, Mao H, Hinds TR, Leyser O, Zheng N. 2018. Structural plasticity of D3–D14 ubiquitin ligase in strigolactone signalling. Nature 563, 652–656. 30464344 10.1038/s41586-018-0743-5 PMC6265067

[CIT0035] Soneson C , Love MI, Robinson MD. 2015. Differential analyses for RNA-seq: transcript-level estimates improve gene-level inferences. F1000Res 4, 1521. 26925227 10.12688/f1000research.7563.1 PMC4712774

[CIT0036] Song X , Lu Z, Yu H, et al. 2017. IPA1 functions as a downstream transcription factor repressed by D53 in strigolactone signaling in rice. Cell Research 27, 1128–1141. 28809396 10.1038/cr.2017.102 PMC5587847

[CIT0037] Sorefan K , Booker J, Haurogné K, et al. 2003. MAX4 and RMS1 are orthologous dioxygenase-like genes that regulate shoot branching in Arabidopsis and pea. Genes and Development 17, 1469–1474. 12815068 10.1101/gad.256603 PMC196077

[CIT0038] Soundappan I , Bennett T, Morffy N, Liang Y, Stanga JP, Abbas A, Leyser O, Nelson DC. 2015. SMAX1-LIKE/D53 family members enable distinct MAX2-dependent responses to strigolactones and karrikins in Arabidopsis. The Plant Cell 27, 3143–3159. 26546447 10.1105/tpc.15.00562 PMC4682302

[CIT0039] Sun H , Guo X, Qi X, Feng F, Xie X, Zhang Y, Zhao Q. 2021. SPL14/17 act downstream of strigolactone signalling to modulate rice root elongation in response to nitrate supply. The Plant Journal 106, 649–660. 33547682 10.1111/tpj.15188

[CIT0040] Sun H , Tao J, Liu S, Huang S, Chen S, Xie X, Yoneyama K, Zhang Y, Xu G. 2014. Strigolactones are involved in phosphate- and nitrate-deficiency-induced root development and auxin transport in rice. Journal of Experimental Botany 65, 6735–6746. 24596173 10.1093/jxb/eru029 PMC4246174

[CIT0041] Takeda T , Suwa Y, Suzuki M, Kitano H, Ueguchi-Tanaka M, Ashikari M, Matsuoka M, Ueguchi C. 2003. The OsTB1 gene negatively regulates lateral branching in rice. The Plant Journal 33, 513–520. 12581309 10.1046/j.1365-313x.2003.01648.x

[CIT0042] Umehara M , Hanada A, Magome H, Takeda-Kamiya N, Yamaguchi S. 2010. Contribution of strigolactones to the inhibition of tiller bud outgrowth under phosphate deficiency in rice. Plant and Cell Physiology 51, 1118–1126. 20542891 10.1093/pcp/pcq084 PMC2900824

[CIT0043] Umehara M , Hanada A, Yoshida S, et al. 2008. Inhibition of shoot branching by new terpenoid plant hormones. Nature 455, 195–200. 18690207 10.1038/nature07272

[CIT0044] Verwoerd TC , Dekker BM, Hoekema A. 1989. A small-scale procedure for the rapid isolation of plant RNAs. Nucleic Acids Research 17, 2362–2362. 2468132 10.1093/nar/17.6.2362 PMC317610

[CIT0045] Vinde MH , Cao D, Chesterfield RJ, et al. 2022. Ancestral sequence reconstruction of the CYP711 family reveals functional divergence in strigolactone biosynthetic enzymes associated with gene duplication events in monocot grasses. New Phytologist 235, 1900–1912. 35644901 10.1111/nph.18285 PMC9544836

[CIT0046] Wakabayashi T , Yasuhara R, Miura K, Takikawa H, Mizutani M, Sugimoto Y. 2021. Specific methylation of (11R)-carlactonoic acid by an Arabidopsis SABATH methyltransferase. Planta 254, 88. 34586497 10.1007/s00425-021-03738-6

[CIT0047] Wang H , Eggert K, Hajirezaei MR, et al. 2018. Abscisic acid influences tillering by modulation of strigolactones in barley. Journal of Experimental Botany 69, 3883–3898. 29982677 10.1093/jxb/ery200 PMC6054196

[CIT0048] Wang J , Lu K, Nie H, Zeng Q, Wu B, Qian J, Fang Z. 2018. Rice nitrate transporter OsNPF7.2 positively regulates tiller number and grain yield. Rice 11, 12. 29484500 10.1186/s12284-018-0205-6 PMC5826914

[CIT0049] Wang L , Wang B, Jiang L, Liu X, Li X, Lu Z, Meng X, Wang Y, Smith SM, Li J. 2015. Strigolactone signaling in Arabidopsis regulates shoot development by targeting D53-Like SMXL repressor proteins for ubiquitination and degradation. The Plant Cell 27, 3128–3142. 26546446 10.1105/tpc.15.00605 PMC4682305

[CIT0050] Wang L , Wang B, Yu H, et al. 2020. Transcriptional regulation of strigolactone signalling in Arabidopsis. Nature 583, 277–281. 32528176 10.1038/s41586-020-2382-x

[CIT0051] Wu S , Li Y. 2021. A unique sulfotransferase-involving strigolactone biosynthetic route in sorghum. Frontiers in Plant Science 12, 793459. 34970291 10.3389/fpls.2021.793459 PMC8713700

[CIT0052] Xiu-mei W , Yue-yang L, Ling LI, et al. 2015. Identification and cloning of tillering-related genes *OsMAX1* in rice. Rice Science 22, 255–263.

[CIT0053] Xu J , Zha M, Li Y, Ding Y, Chen L, Ding C, Wang S. 2015. The interaction between nitrogen availability and auxin, cytokinin, and strigolactone in the control of shoot branching in rice (*Oryza sativa* L.). Plant Cell Reports 34, 1647–1662. 26024762 10.1007/s00299-015-1815-8

[CIT0054] Yao R , Ming Z, Yan L, et al. 2016. DWARF14 is a non-canonical hormone receptor for strigolactone. Nature 536, 469–473. 27479325 10.1038/nature19073

[CIT0055] Yoneyama K , Akiyama K, Brewer PB, et al. 2020a. Hydroxyl carlactone derivatives are predominant strigolactones in Arabidopsis. Plant Direct 4, e00219. 32399509 10.1002/pld3.219 PMC7207163

[CIT0056] Yoneyama K , Brewer PB. 2021. Strigolactones, how are they synthesized to regulate plant growth and development? Current Opinion in Plant Biology 63, 102072. 34198192 10.1016/j.pbi.2021.102072

[CIT0057] Yoneyama K , Kisugi T, Xie X, Arakawa R, Ezawa T, Nomura T, Yoneyama K. 2015. Shoot-derived signals other than auxin are involved in systemic regulation of strigolactone production in roots. Planta 241, 687–698. 25417194 10.1007/s00425-014-2208-x

[CIT0058] Yoneyama K , Mori N, Sato T, et al. 2018. Conversion of carlactone to carlactonoic acid is a conserved function of MAX1 homologs in strigolactone biosynthesis. New Phytologist 218, 1522–1533. 29479714 10.1111/nph.15055

[CIT0059] Yoneyama K , Xie X, Kim HI, Kisugi T, Nomura T, Sekimoto H, Yokota T, Yoneyama K. 2012. How do nitrogen and phosphorus deficiencies affect strigolactone production and exudation? Planta 235, 1197–1207. 22183123 10.1007/s00425-011-1568-8 PMC3362704

[CIT0060] Yoneyama K , Xie X, Kusumoto D, Sekimoto H, Sugimoto Y, Takeuchi Y, Yoneyama K. 2007a. Nitrogen deficiency as well as phosphorus deficiency in sorghum promotes the production and exudation of 5-deoxystrigol, the host recognition signal for arbuscular mycorrhizal fungi and root parasites. Planta 227, 125–132. 17684758 10.1007/s00425-007-0600-5

[CIT0061] Yoneyama K , Xie X, Nomura T, Yoneyama K. 2020b. Do phosphate and cytokinin interact to regulate strigolactone biosynthesis or act independently? Frontiers in Plant Science 11, 438. 32508849 10.3389/fpls.2020.00438 PMC7251057

[CIT0062] Yoneyama K , Yoneyama K, Takeuchi Y, Sekimoto H. 2007b. Phosphorus deficiency in red clover promotes exudation of orobanchol, the signal for mycorrhizal symbionts and germination stimulant for root parasites. Planta 225, 1031–1038. 17260144 10.1007/s00425-006-0410-1

[CIT0063] Zhang Y , van Dijk ADJ, Scaffidi A, et al. 2014. Rice cytochrome P450 MAX1 homologs catalyze distinct steps in strigolactone biosynthesis. Nature Chemical Biology 10, 1028–1033. 25344813 10.1038/nchembio.1660

[CIT0064] Zhou F , Lin Q, Zhu L, et al. 2013. D14–SCFD3-dependent degradation of D53 regulates strigolactone signalling. Nature 504, 406–410. 24336215 10.1038/nature12878 PMC4096652

