# Supplementary material for: Nutritional and tissue-specific regulation of cytochrome P450 CYP711A MAX1 homologues and strigolactone biosynthesis in wheat
Source: J Exp Bot. 2023 Jan 10;74(6):1890–910. doi: 10.1093/jxb/erad008 (PMC10049918; doi:10.1093/jxb/erad008)
Supplement: erad008_suppl_Supplementary_Figures_S1-S11 [file erad008_suppl_supplementary_figures_s1-s11.pdf]

Supplementary Figures

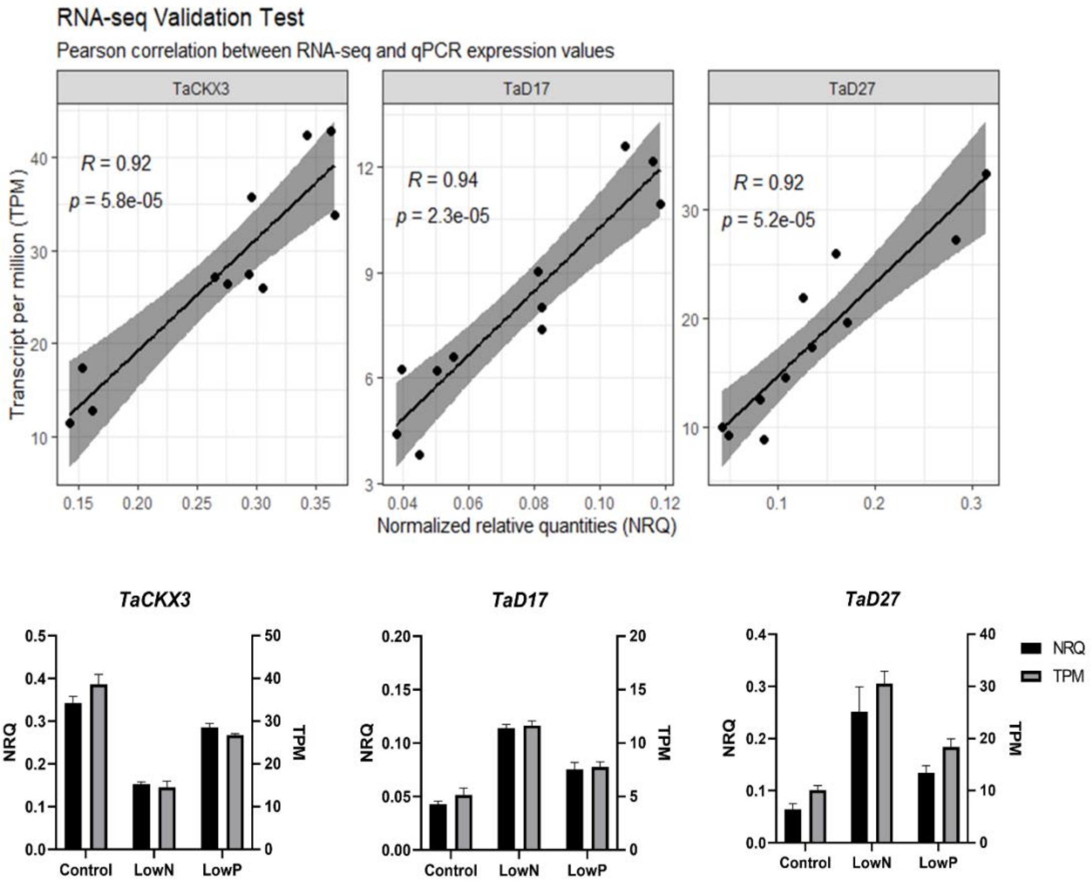

Fig. S1. RNA-seq validation test results. Three genes were included in the validation test (*TaCKX3*, *TaD17* and *TaD27*). **(Top)** Pearson correlation analysis of TPM values obtained from the RNA-seq and NRQ expression values obtained from RT-qPCR. **(Bottom)** Comparison between average treatment effects (n=4) based on RNA-seq (TPM) and RT-qPCR data (NRQ).

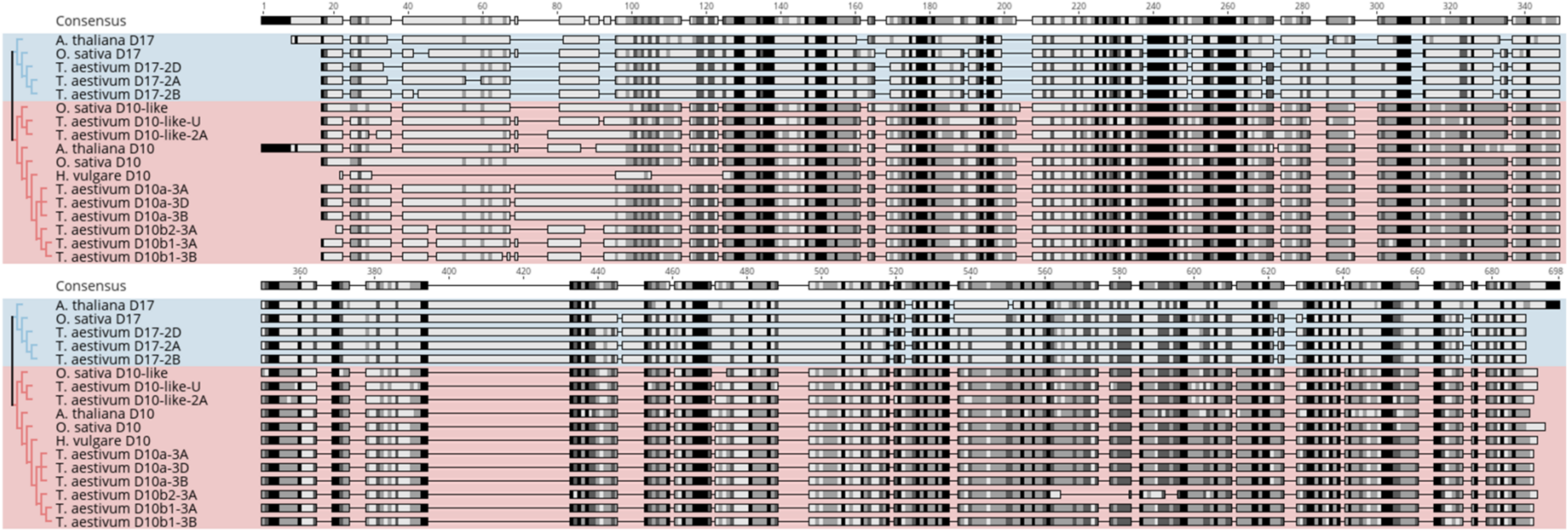

Fig. S2. Phylogenetic relationship and protein alignment of D17/CCD7 (in blue) and D10/CCD8 (in red) proteins from hexaploid wheat, rice, barley and Arabidopsis. The tree was constructed using MUSCLE sequence alignment and the neighbour-joining method. TraesCS2A02G414600, TraesCS2B02G433800 and TraesCS2D02G411900 were assigned as *TaD17* homoeologues. TraesCS3A02G274300, TraesCS3B02G308000, TraesCS3D02G273500 were assigned as *TaD10a* and based on publicly available gene expression data **(Fig. S3)** and were considered as functional orthologues of D10 in wheat.

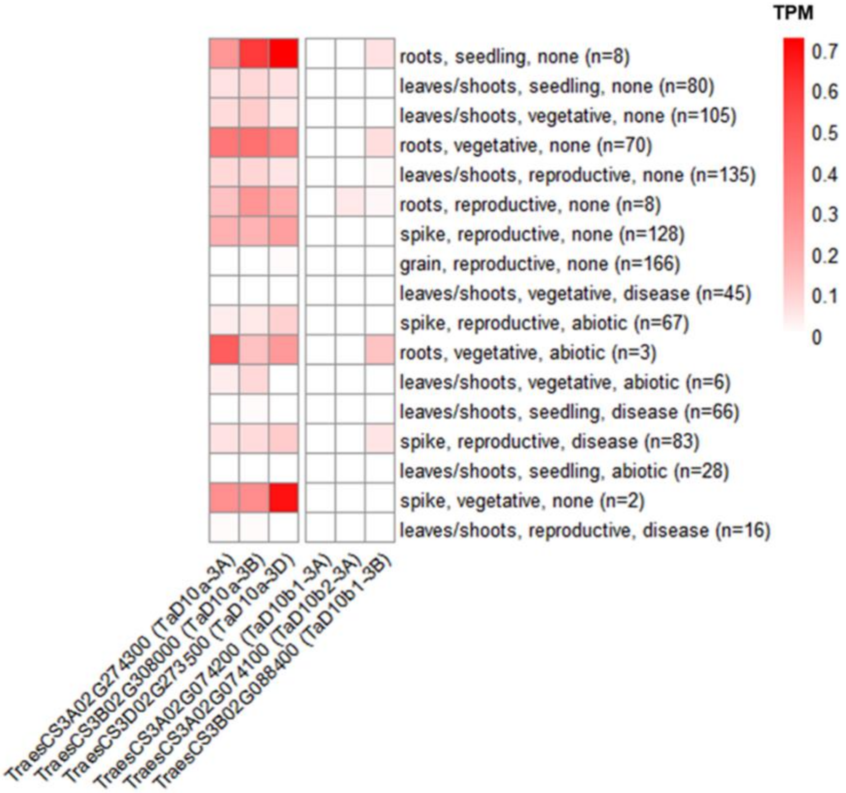

Fig. S3. Heatmap of putative *TaD10* expression profiles in different tissues and under different conditions extracted from expVIP Wheat expression Browser (Ramírez-González et al., 2018).

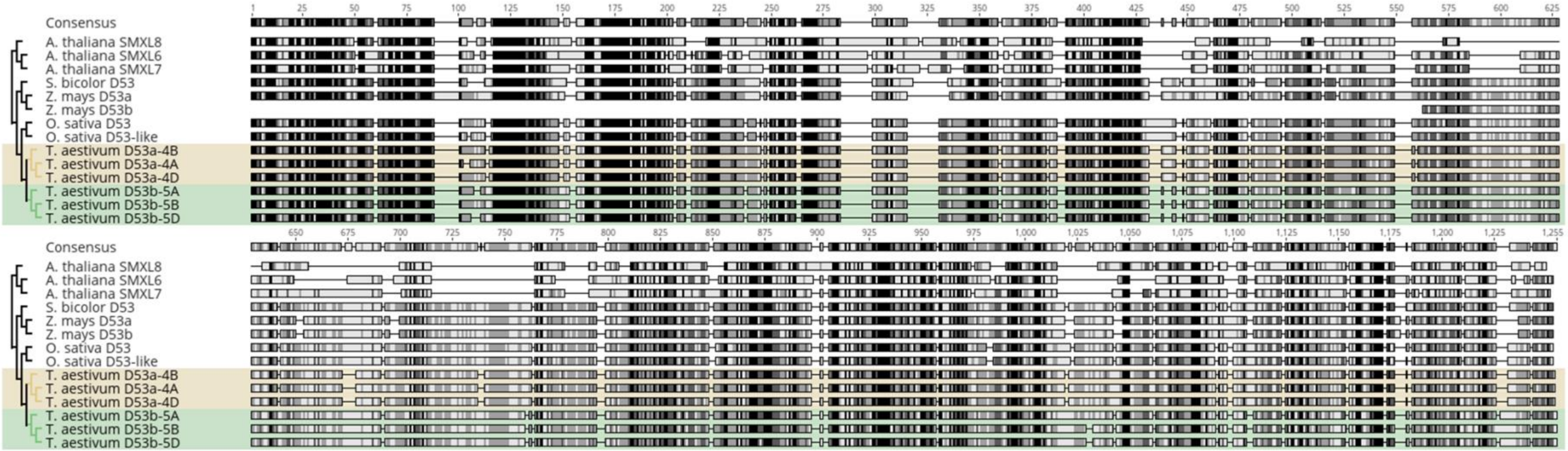

Fig. S4. Protein alignment and phylogenetic relationship of D53 proteins from hexaploid wheat, rice, sorghum, maize and Arabidopsis. The tree was constructed using MUSCLE sequence alignment and the neighbour-joining method. TraesCS4A02G182800, TraesCS4B02G135800 and TraesCS4D02G130600 were assigned as *TaD53a* homoeologues (in yellow), and TraesCS5A02G155000, TraesCS5B02G153200 and TraesCS5D02G159900 were assigned as *TaD53b* (in green).

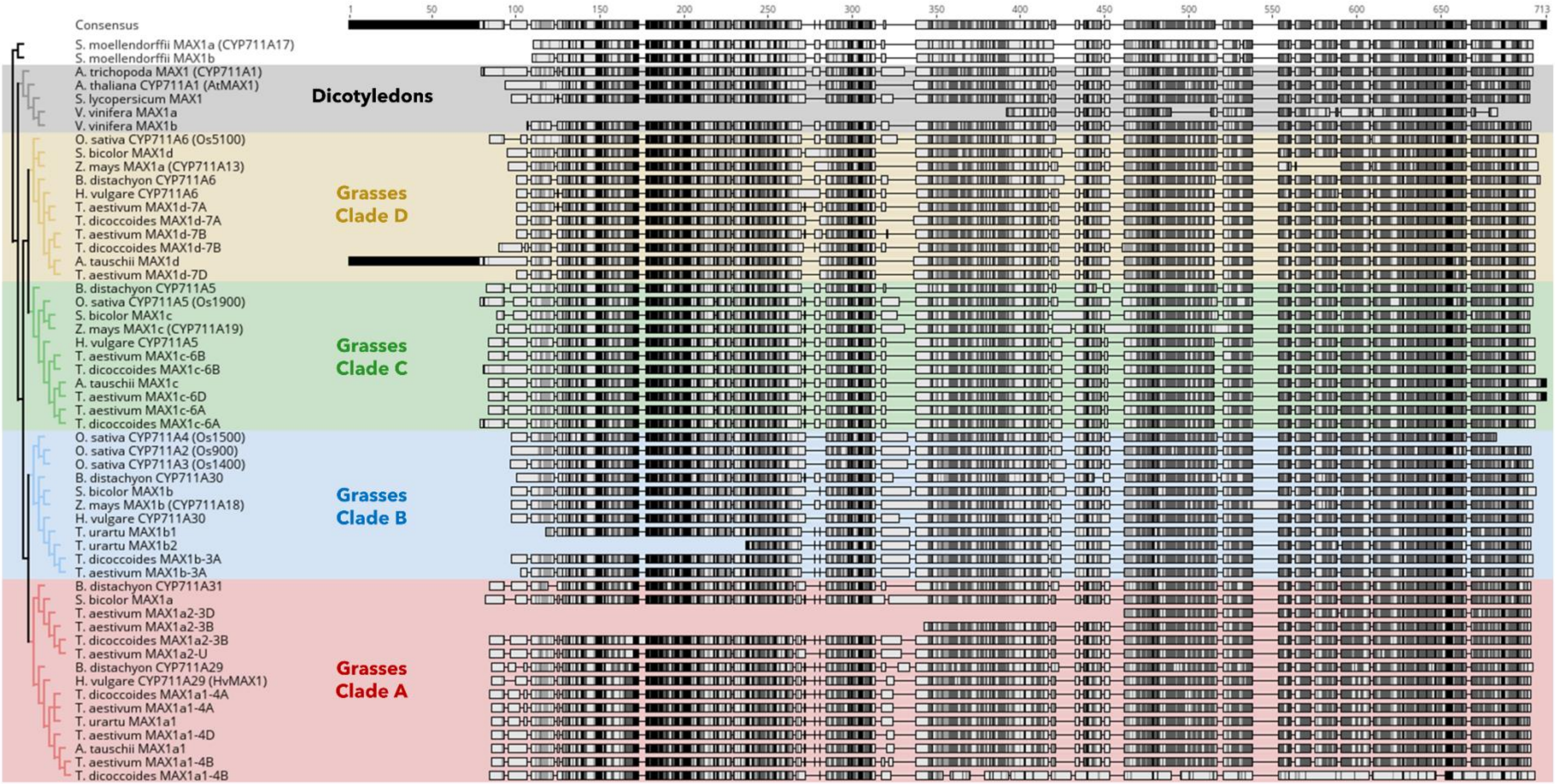

Fig. S5. Protein alignment and phylogenetic relationship of CYP711A/MAX1 proteins mainly from grasses such as hexaploid wheat, rice, barley, sorghum, maize, *B. distachyon*, from dicotyledons such as Arabidopsis, *Solanum lycopersicum*, *Vitis vinifera* and *Amborella trichopoda* and the lycophyte, *Selaginella moellendorffii*. The protein sequences of wheat progenitors; *Triticum urartu* (AA), *Triticum dicoccoides* (AABB) and *Aegilops tauschii* (DD) were also included. The tree was constructed using MUSCLE sequence alignment and the neighbour-joining method. The different colours (red, blue, green and yellow) correspond to the four different clades of CYP711A/MAX1 identified in grasses (A-D). The accession number of the sequences used in the analysis can be found in **Table S5**.

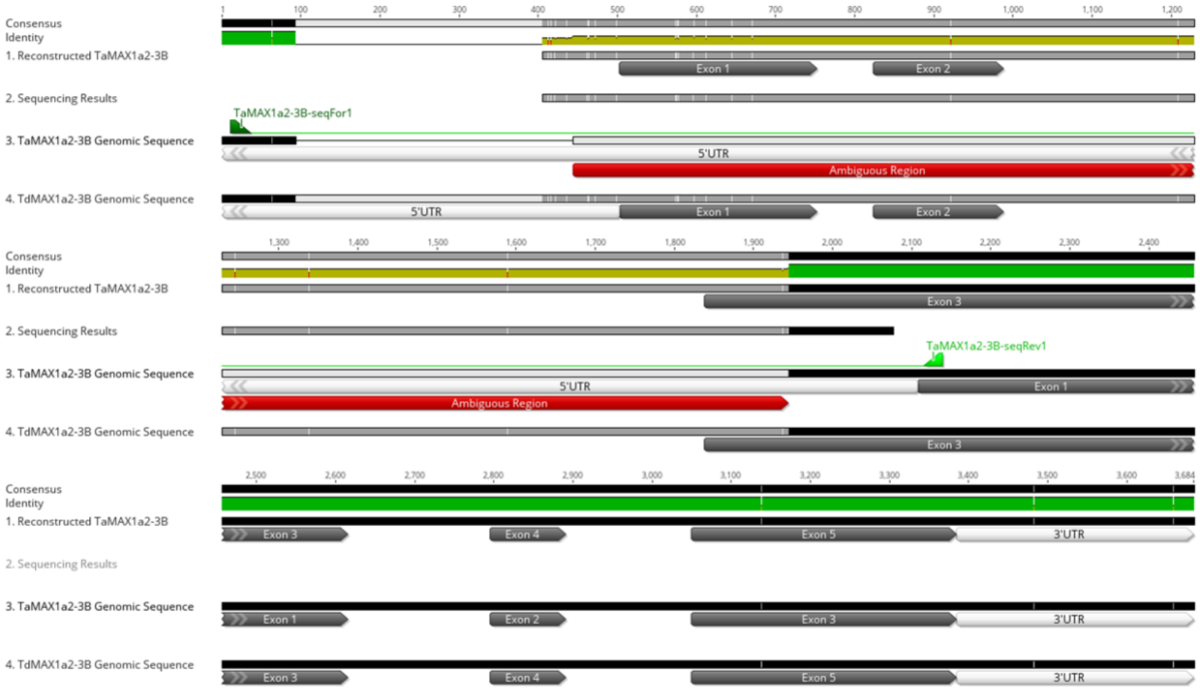

Fig. S6. Nucleotide sequence alignment of hexaploid wheat (*Ta*) and *T. dicoccoides* (*Td*) *MAX1a2-3B* genomic sequences. The annotated *TaMAX1a2-3B* (TraesCS3B02G088700) contains only three exons (grey annotations), whereas *TdMAX1a2-3B* (TRIDC3BG012120) contains five exons. An ambiguous region of >1.5 kb was found upstream of *TaMAX1a2-3B* (red annotation). Sequencing of the region upstream of *TaMAX1a2-3B* showed that the ambiguous region is homologous to *TdMAX1a2-3B*, therefore *TaMAX1a2-3B* also contains five exons encoding a functional CYP711A/MAX1 homologue.

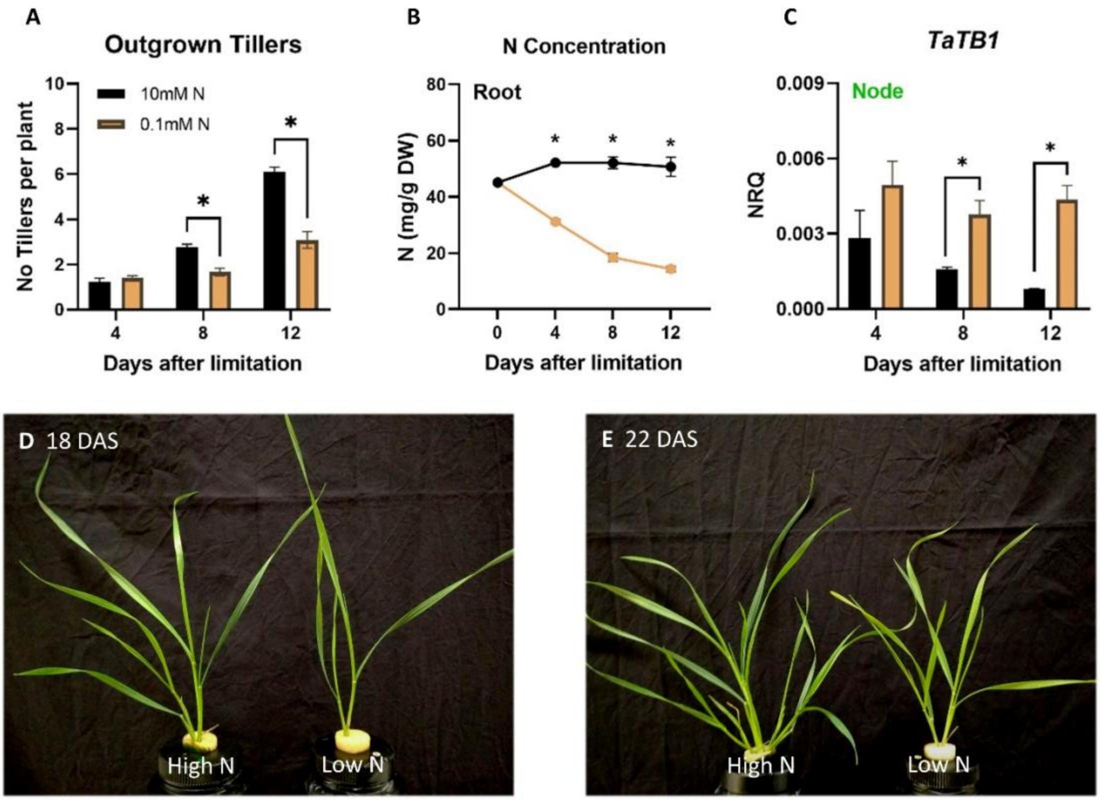

Fig. S7. Time-course analysis of the effect of N limitation on (A) tillering, (B) root N concentration and (C) gene expression levels of *TaTB1* in the basal nodes of wheat (cv Cadenza) 0, 4, 8 and 12 days after N limitation. Values are means of three biological replicates and error bars represent SE. * denotes a statistically significant difference in the gene expression levels between low N and high N plants at each time point based on Fisher's LSD test. **(D, E) Representative wheat plants (cv Cadenza) grown under high N and low N conditions at 8 days (D, 18 DAS) and 12 (E, 22 DAS) days after N limitation.**

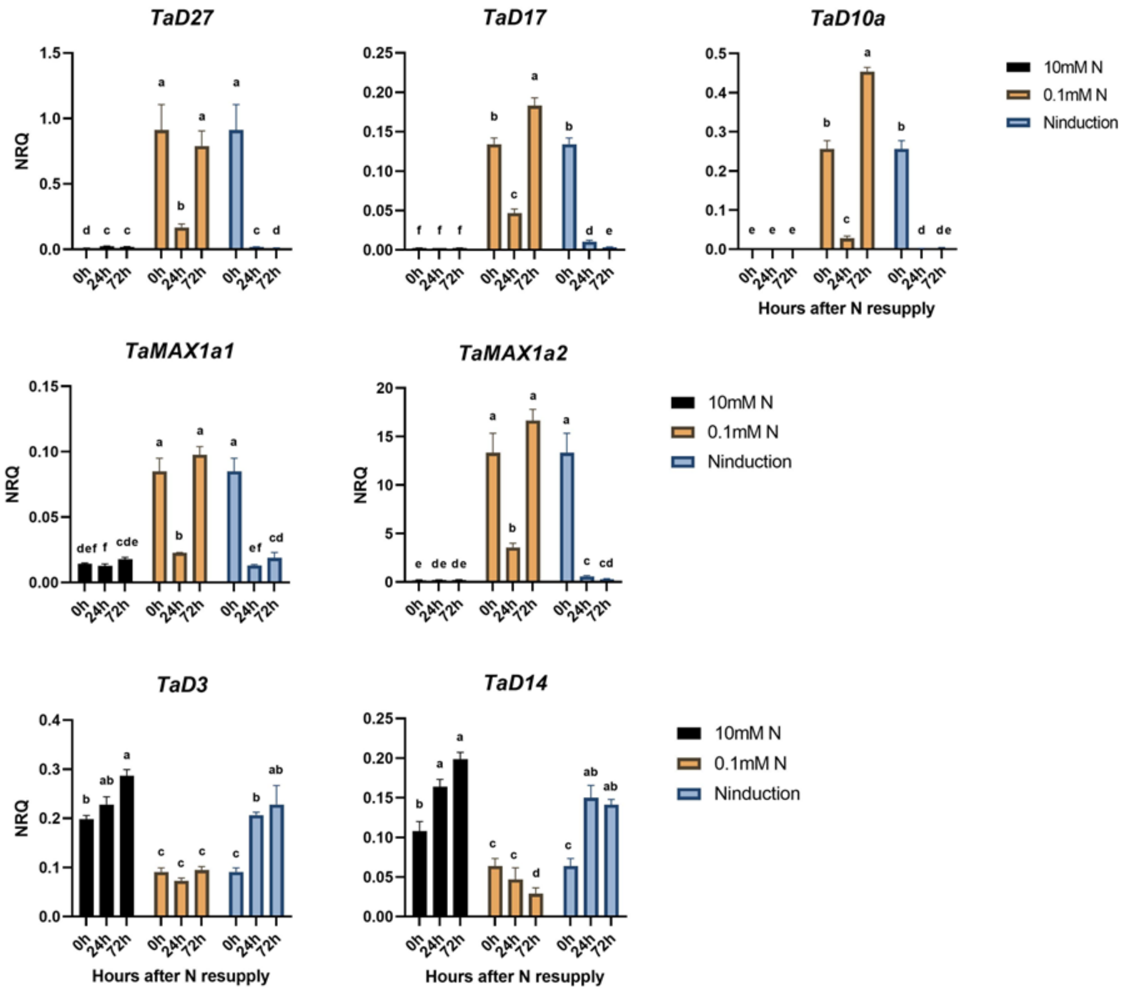

Fig. S8. Time-course analysis of the gene expression levels of SL biosynthetic (*TaD27*, *TaD17*, *TaD10a*, *TaMAX1a1* and *TaMAX1a2*) and perception genes (*TaD3* and *TaD14*) in the root of wheat (cv Cadenza) before (0h), 24h and 72h after N resupply to N-limited plants. Plants were resupplied with N 8 days after N-limitation. Values are means of three biological replicates and error bars represent SE. Statistical analysis was conducted with an unbalanced 2-way ANOVA in log2(1/NRQ) transformed values. Different letters denote statistically significant differences in the gene expression levels between the means based on Fisher's LSD test.

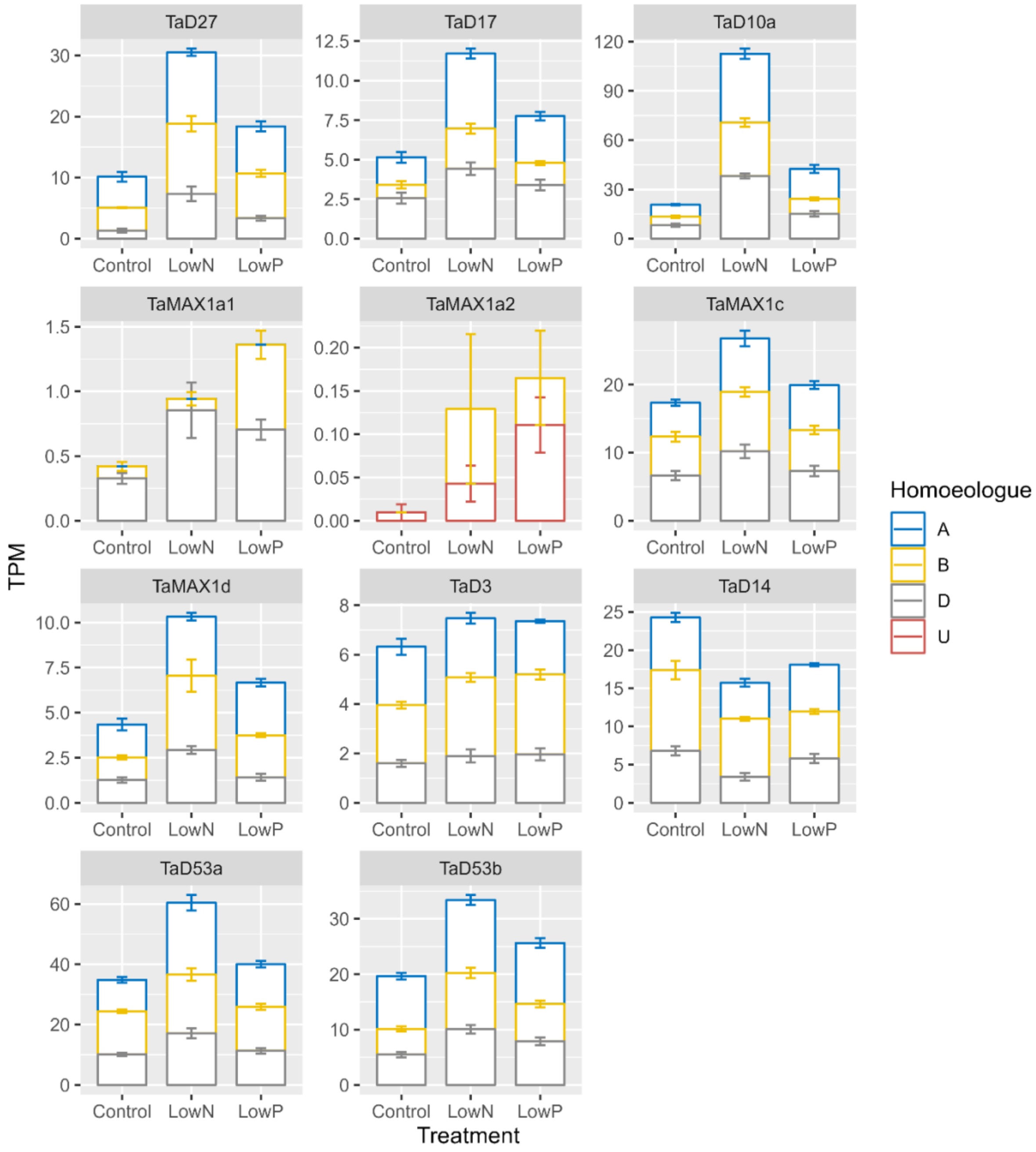

Fig. S9. Transcript abundance of SL biosynthetic (*TaD27*, *TaD17*, *TaD10a*, *TaMAX1a1*, *TaMAX1a2*, *TaMAX1c* and *TaMAX1d*), perception (*TaD3* and *TaD14*) and signalling (*TaD53a* and *TaD53b*) genes and their homoeologues in the basal nodes of wheat (cv Cadenza) grown under N and P limitation for 8 days based on the RNA-seq data. Plants were introduced to nutrient limitation at 10 DAS. Values are means of four biological replicates and error bars represent SE. A, B, D and U represent A, B, D and U homoeologue expression data.

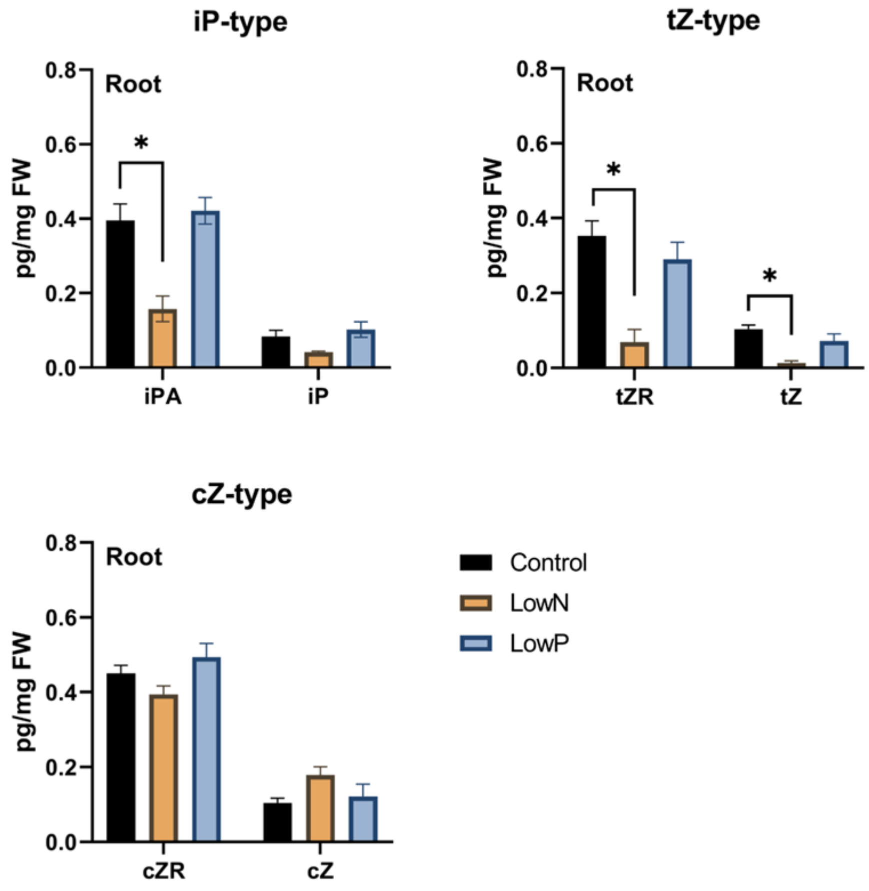

Fig. S10. Concentration of different CKs (pg/mg) in the root of wheat (cv Cadenza) grown under Control (10 mM N and 1 mM P), Low N (0.1 mM N) and Low P (0.01 mM P) conditions for 8 days. Plants were introduced to nutrient limitation at 10 DAS. Data are means ± SE (n=4). Statistical analysis was conducted with ANOVA. * denotes statistically significant difference based on Fisher's LSD test. iPA: isopentenyl adenosine; iP: isopentenyl adenine; tZ: trans-zeatin, tZR: tZ-riboside; cZ: cis-zeatin; cZR: cZ-riboside.

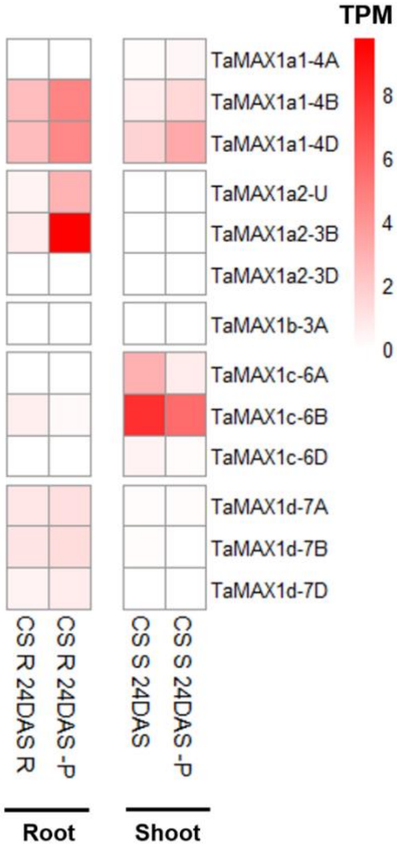

Fig. S11. Heatmap comparison of *TaMAX1* gene expression values expressed as TPM in root and shoot of P-sufficient and P-stressed wheat plants. Data are means of at least three biological replicates. Data were retrieved from Oono et al., 2013 (BioProject: PRJDB2496, SRA: DRP000768). CS: cv Chinese Spring.
